# Code Generation in Computational Neuroscience: A Review of Tools and Techniques

**DOI:** 10.3389/fninf.2018.00068

**Published:** 2018-11-05

**Authors:** Inga Blundell, Romain Brette, Thomas A. Cleland, Thomas G. Close, Daniel Coca, Andrew P. Davison, Sandra Diaz-Pier, Carlos Fernandez Musoles, Padraig Gleeson, Dan F. M. Goodman, Michael Hines, Michael W. Hopkins, Pramod Kumbhar, David R. Lester, Bóris Marin, Abigail Morrison, Eric Müller, Thomas Nowotny, Alexander Peyser, Dimitri Plotnikov, Paul Richmond, Andrew Rowley, Bernhard Rumpe, Marcel Stimberg, Alan B. Stokes, Adam Tomkins, Guido Trensch, Marmaduke Woodman, Jochen Martin Eppler

**Affiliations:** ^1^Forschungszentrum Jülich, Institute of Neuroscience and Medicine (INM-6), Institute for Advanced Simulation (IAS-6), JARA BRAIN Institute I, Jülich, Germany; ^2^Sorbonne Université, INSERM, CNRS, Institut de la Vision, Paris, France; ^3^Department of Psychology, Cornell University, Ithaca, NY, United States; ^4^Monash Biomedical Imaging, Monash University, Melbourne, VIC, Australia; ^5^Department of Automatic Control and Systems Engineering, University of Sheffield, Sheffield, United Kingdom; ^6^Unité de Neurosciences, Information et Complexité, CNRS FRE 3693, Gif sur Yvette, France; ^7^Forschungszentrum Jülich, Simulation Lab Neuroscience, Jülich Supercomputing Centre, Institute for Advanced Simulation, Jülich Aachen Research Alliance, Jülich, Germany; ^8^Department of Neuroscience, Physiology and Pharmacology, University College London, London, United Kingdom; ^9^Department of Electrical and Electronic Engineering, Imperial College London, London, United Kingdom; ^10^Department of Neurobiology, School of Medicine, Yale University, New Haven, CT, United States; ^11^Advanced Processor Technologies Group, School of Computer Science University of Manchester, Manchester, United Kingdom; ^12^Blue Brain Project, EPFL Campus Biotech, Geneva, Switzerland; ^13^Centro de Matemática, Computação e Cognição Universidade Federal do ABC, São Bernardo do Campo, Brazil; ^14^Faculty of Psychology, Institute of Cognitive Neuroscience Ruhr-University Bochum, Bochum, Germany; ^15^Kirchhoff-Institute for Physics Universität Heidelberg, Heidelberg, Germany; ^16^Centre for Computational Neuroscience and Robotics, School of Engineering and Informatics University of Sussex, Brighton, United Kingdom; ^17^RWTH Aachen University, Software Engineering Jülich Aachen Research Alliance, Aachen, Germany; ^18^Department of Computer Science University of Sheffield, Sheffield, United Kingdom; ^19^Institut de Neurosciences des Systèmes Aix Marseille Université, Marseille, France

**Keywords:** code generation, simulation, neuronal networks, domain specific language, modeling language

## Abstract

Advances in experimental techniques and computational power allowing researchers to gather anatomical and electrophysiological data at unprecedented levels of detail have fostered the development of increasingly complex models in computational neuroscience. Large-scale, biophysically detailed cell models pose a particular set of computational challenges, and this has led to the development of a number of domain-specific simulators. At the other level of detail, the ever growing variety of point neuron models increases the implementation barrier even for those based on the relatively simple integrate-and-fire neuron model. Independently of the model complexity, all modeling methods crucially depend on an efficient and accurate transformation of mathematical model descriptions into efficiently executable code. Neuroscientists usually publish model descriptions in terms of the mathematical equations underlying them. However, actually simulating them requires they be translated into code. This can cause problems because errors may be introduced if this process is carried out by hand, and code written by neuroscientists may not be very computationally efficient. Furthermore, the translated code might be generated for different hardware platforms, operating system variants or even written in different languages and thus cannot easily be combined or even compared. Two main approaches to addressing this issues have been followed. The first is to limit users to a fixed set of optimized models, which limits flexibility. The second is to allow model definitions in a high level interpreted language, although this may limit performance. Recently, a third approach has become increasingly popular: using code generation to automatically translate high level descriptions into efficient low level code to combine the best of previous approaches. This approach also greatly enriches efforts to standardize simulator-independent model description languages. In the past few years, a number of code generation pipelines have been developed in the computational neuroscience community, which differ considerably in aim, scope and functionality. This article provides an overview of existing pipelines currently used within the community and contrasts their capabilities and the technologies and concepts behind them.

## 1. Introduction

All brains are composed of a huge variety of neuron and synapse types. In computational neuroscience we use models for mimicking the behavior of these elements and to gain an understanding of the brain's behavior by conducting *simulation experiments* in *neural simulators*. These models are usually defined by a set of variables which have either concrete values or use functions and differential equations that describe the temporal evolution of the variables.

A simple but instructive example is the integrate-and-fire neuron model, which describes the dynamics of the membrane potential *V* in the following way: when *V* is below the *spiking threshold* θ, which is typically at around −50 mV, the time evolution is governed by a differential equation of the type:

$$\begin{array}{l} \frac{d}{dt}V=f\left(V\right) \end{array}$$

where *f* is a function that is possibly non-linear.

Once *V* reaches its threshold θ, a *spike* is fired and *V* is set back to *E*_L_ for a certain time called the *refractory period*. *E*_L_ is called the *resting potential* and is typically around −70 mV. After this time the evolution of the equation starts again. An important simplification compared to biology is that the exact course of the membrane potential during the spike is either completely neglected or only partially considered in most models. Threshold detection is rather added algorithmically on top of the modeled subthreshold dynamics.

Two of the most common variants of this type of model are the *current-based* and the *conductance-based* integrate-and-fire models. For the case of the current-based model we have the following general form:

$$\begin{array}{ll} \frac{d}{dt}V\left(t\right) & =\frac{1}{\tau }\left(E_{\text{L}}-V\left(t\right)\right)\\ & \;+\frac{1}{C}I\left(t\right)+F\left(V\left(t\right)\right). \end{array}$$

Here *C* is the *membrane capacitance*, τ the *membrane time constant*, and *I* the *input current* to the neuron. Assuming that spikes will be fixed to temporal grid points, *I*(*t*) is the sum of currents generated by all incoming spikes at all grid points in time *t*_*i*_ ≤ *t* scaled by their synaptic weight plus a piecewise constant function *I*_ext_ that models an external input:

$$\begin{array}{l} I\left(t\right)=\underset{i\in \mathbb{N},t_{i}\leq t}{\sum }\underset{k\in S_{t_{i}}}{\sum }I_{k}\left(t-t_{i}\right)+I_{\text{ext}}\left(t\right) \end{array}$$

*S*_*t*_ is the set of synapses that deliver a spike to the neuron at time *t* and *I*_*k*_ is the current that enters the neuron through synapse *k*. *F* is some non-linear function of *V* that may be zero.

One concrete example is the simple integrate-and-fire neuron with alpha-shaped synaptic input, where *F*(*V*)≡0, $I_{k}\left(t\right)=\frac{e}{\tau_{syn}}te^{-t/\tau_{\text{syn}}}$ and τ_syn_ is the rise time, which is typically around 0.2–2.0 ms.

When implementing such models in neural simulators their differential equations must be solved as part of the *neuron model implementation*. One typical approach is to use a numeric integrator, e.g., a simple Euler method.

For a simulation stepsize *h* and some given approximation *V*_*t*_ of *V*(*t*), using an Euler method would lead to the following approximation *V*_*t*+*h*_ of *V*(*t*+*h*):

$$\begin{array}{l} V_{t+h}=V_{t}+h\left(\frac{1}{\tau }\left(E_{\text{L}}-V_{t}\right)+\frac{1}{C}I\left(t\right)\right). \end{array}$$

Publications in computational neuroscience mostly contain descriptions of models in terms of their mathematical equations and the algorithms to add additional behavior such as the mechanism for threshold detection and spike generation. However, if looking at a model implementation and comparing it to the corresponding published model description, one often finds that they are not in agreement due to the complexity and variety of available forms of abstractions of such a transformation (e.g., Manninen et al., 2017, 2018). Using a general purpose programming language to express the model implementation even aggravates this problem as such languages provide full freedom for model developers while lacking the means to guide them in their challenging task due to the absence of neuroscience domain concepts.

Furthermore, the complexity of the brain enforces the use of a heterogeneous set of models on different abstraction levels that, however, need to efficiently cooperate upon execution. Model compositionality is needed on the abstract mathematical side as well as on the implementation level.

The use of problem-tailored model description languages and standardized simulators is often seen as a way out of the dilemma as they can provide the domain-specificity missing in a general programming language, however often at the cost of restricting the users in their freedom to express arbitrary algorithms.

In other words, engineering complex software systems introduces a conceptual gap between problem domains and solution domains. Model driven development (MDD; France and Rumpe, 2007) aims at closing this gap by using abstract models for the description of domain problems and code generation for creating executable software systems (Kleppe et al., 2003). Early MDD techniques have been already successfully applied in computer science for decades (Davis et al., 2006). These techniques ensure reduced development costs and increased software quality of resulting software systems (Van Deursen and Klint, 1998; Fieber et al., 2008; Stahl et al., 2012). MDD also provides methodological concepts to increase design and development speed of simulation code.

It turns out that MDD is not restricted to the software engineering domain, but can be applied in many science and also engineering domains (Harel, 2005; Topcu et al., 2016). For example, the Systems Biology Markup Language (SBML; Hucka et al., 2003) from the domain of biochemistry enables modeling of biochemical reaction networks, like cell signaling pathways, metabolic pathways, and gene regulation, and has several software tools that support users with the creation, import, export, simulation, and further processing of models expressed in SBML.

MDD works best if the underlying modeling language fits to the problem domain and thus is specifically engineered for that domain (Combemale et al., 2016). The modeling language must provide modularity in several domains: individual neurons of different behavior must be modeled, time, and geometric abstractions should be available, composition of neurons to large networks must be possible and reuse of neuron models or neuron model fragments must be facilitated.

In the context of computational neuroscience (Churchland et al., 1993) the goal of MDD is to transform complex and abstract mathematical neuron, synapse, and network specifications into efficient platform-specific executable representations. There is no lack of neural simulation environments that are able to simulate models efficiently and accurately, each specializing on networks of different size and complexity. Some of these simulators (e.g., NEST, Gewaltig and Diesmann 2007) have included optimized neural and synaptic models written in low-level code without support for more abstract, mathematical descriptions. Others (e.g., NEURON with NMODL, Hines and Carnevale, 1997, see section 2.7) have provided a separate model description language together with tools to convert these descriptions into reusable model components. Recently, such support has also been added to the NEST simulator via NESTML (Plotnikov et al., 2016, see section 2.4). Finally, other simulators (e.g., Brian, Goodman 2010, see section 2.1; The Virtual Brain, see section 2.10) include model descriptions as integral parts of the simulation script, transparently converting these descriptions into executable code.

These approaches to model descriptions have been complemented in recent years by various initiatives creating simulator-independent model description languages. These languages completely separate the model description from the simulation environment and are therefore not directly executable. Instead, they provide code generation tools to convert the descriptions into code for target environments such as the ones mentioned above, but also for more specialized target platforms such as GPUs (e.g., GeNN, Yavuz et al., 2016, see section 2.2), or neuromorphic chips like SpiNNaker or the BrainScaleS System (see section 3). Prominent description languages include NineML (Raikov et al., 2011, see section 2.6), NeuroML (Goddard et al., 2001; Gleeson et al., 2010), and LEMS (Cannon et al., 2014). These languages are often organized hierarchically, for example LEMS is the low-level description language for neural and synaptic models that can be assembled into a network with a NeuroML description (see section 2.5). Another recently developed description language, SpineML (Richmond et al. 2014, see section 2.8) builds upon LEMS descriptions as well.

A new generation of centralized collaboration platforms like Open Source Brain and the Human Brain Project Collaboratory (see section 3) are being developed to allow greater access to neuronal models for both computationally proficient and non-computational members of the neuroscience community. Here, code generation systems can serve as a means to free the user from installing their own software while still giving them the possibility to create and use their own neuron and synapse models.

This article summarizes the state of the art of code generation in the field of computational neuroscience. In section 2, we introduce some of the most important modeling languages and their code generation frameworks. To ease a comparison of the different technologies employed, each of the sections follows the same basic structure. Section 3 describes the main target platforms for the generation pipelines and introduces the ideas behind the web-based collaboration platforms that are now becoming available to researchers in the field. We conclude by summarizing the main features of the available code generation systems in section 4.

## 2. Tools and code generation pipelines

### 2.1. Brian

**Figure 1:**
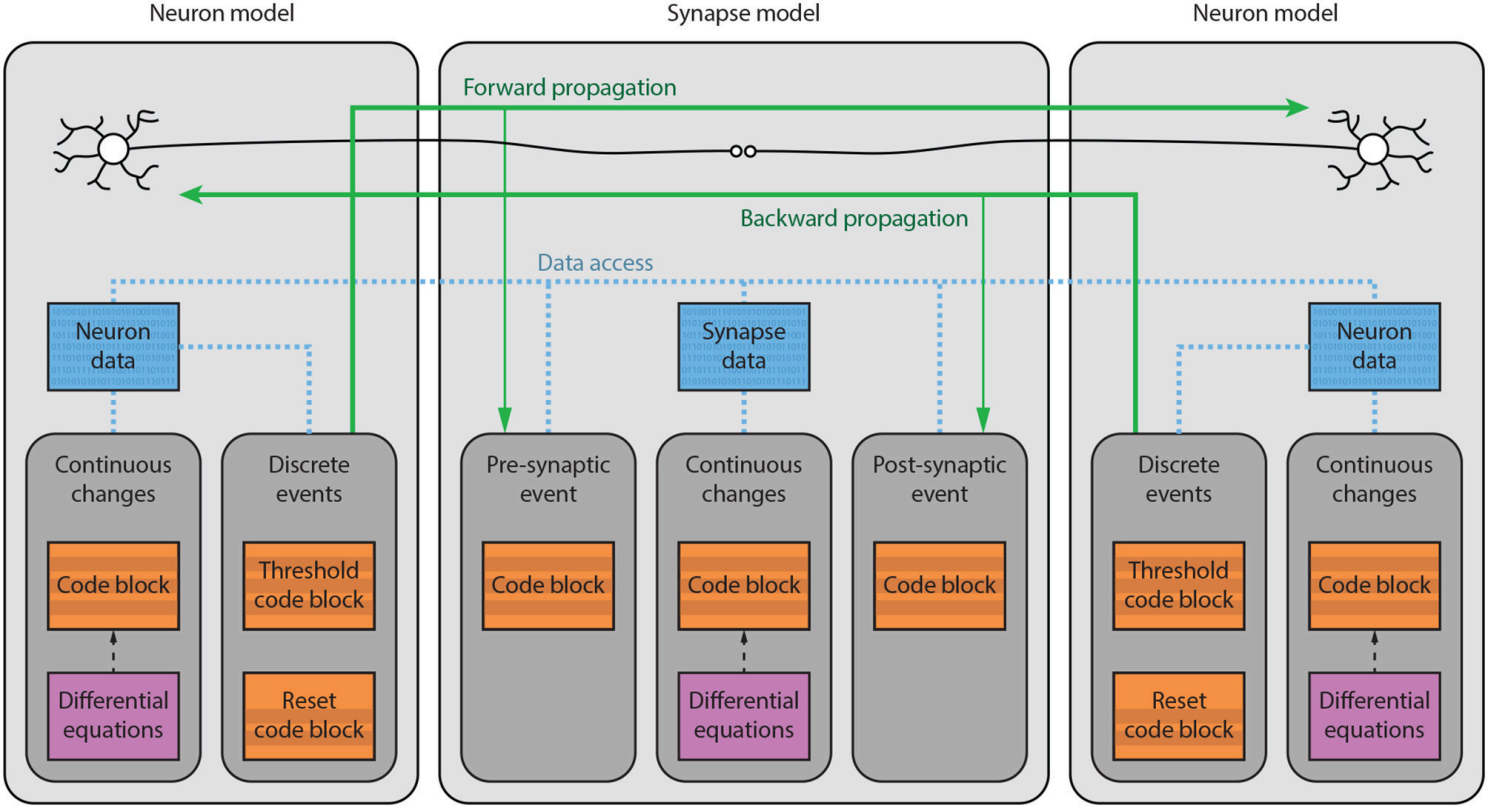
Brian model structure. Brian users define models by specifying equations governing a single neuron or synapse. Simulations consist of an ordered sequence of operations (code blocks) acting on neuronal or synaptic data. A neuronal code block can only modify its own data, whereas a synaptic code block can also modify data from its pre- or post-synaptic neurons. Neurons have three code blocks: one for its continuous evolution (numerical integration), one for checking threshold conditions and emitting spike events, and one for post-spike reset in response to those events. Synapses have three code blocks: two event-based blocks for responding to pre- or postsynaptic spikes (corresponding to forward or backward propagation), and one continuous evolution block. Code blocks can be provided directly, or can be generated from pseudo-code or differential equations.

All versions of the Brian simulator have used code generation, from the simple pure Python code generation for some model components in its earliest versions (Goodman and Brette, 2008, 2009), through the mixed Python/C++ code generation in later versions (Goodman, 2010), to the exhaustive framework in its latest version (2.x) that will be described here. It now uses a consistent code generation framework for all model components, and allows for multiple target languages and devices (Stimberg et al., 2012–2018a, 2014). Brian 2 had code generation as a major design goal, and so the *user model, data model*, and *execution model* were created with this in mind (Figure 1).

#### 2.1.1. Main modeling focus

Brian focuses on modeling networks of point neurons, where groups of neurons are described by the same set of equations (but possibly differ in their parameters). Depending on the equations, such models can range from variants of the integrate-and-fire model to biologically detailed models incorporating a description of multiple ion channels. The same equation framework can also be used to model synaptic dynamics (e.g., short- and long-term plasticity) or spatially extended, multi-compartmental neurons.

#### 2.1.2. Model notation

From the user point of view, the simulation consists of components such as neurons and synapses, each of which are defined by equations given in standard mathematical notation. For example, a leaky integrate-and-fire neuron evolves over time according to the differential equation d*v*/dt = −*v*/τ. In Brian this would be written as the Python string 'dv/dt=-v/tau : volt' in which the part after the colon defines the physical dimensions of the variable *v*. All variables and constants have physical dimensions, and as part of the code generation framework, all operations are checked for dimensional consistency.

Since all aspects of the behavior of a model are determined by user-specified equations, this system offers the flexibility for implementing both standard and non-standard models. For example, the effect of a spike arriving at a synapse is often modeled by an equation such as *v*_post_←*v*_post_+*w* where *v*_post_ is the postsynaptic membrane potential and *w* is a synaptic weight. In Brian this would be rendered as part of the definition of synapses as (…, on_pre='v_post += w'). However, the user could as well also change the value of synaptic or presynaptic neuronal variables. For the example of STDP, this might be something like Synapses(…, on_pre='v_post+=w; Am+=dAm; w=clip(w+Ap, 0, wmax)'), where Am and Ap are synaptic variables used to keep a trace of the pre- and post-synaptic activity, and clip(x, y, z) is a pre-defined function (equivalent to the NumPy function of the same name) that returns x if it is between y and z, or y or z if it is outside this range.

#### 2.1.3. Code generation pipeline

**Figure 2:**
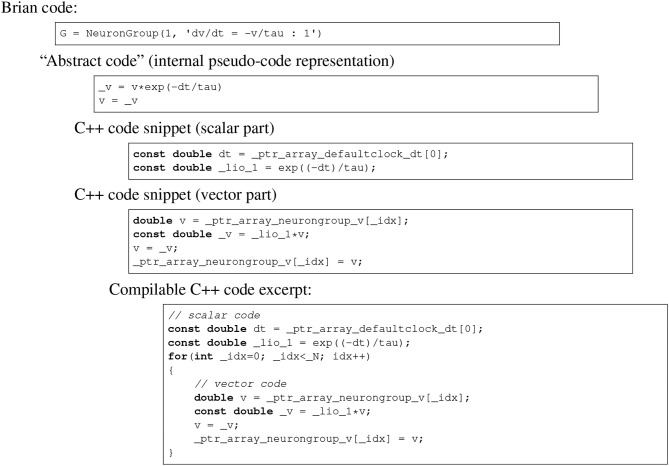
Brian code generation pipeline. Code is transformed in multiple stages: the original Brian code (in Python), with a differential equation given in standard mathematical form; the internal pseudocode or “abstract code” representation (Python syntax), in this case an exact numerical solver for the equations; the C++ code snippets generated from the abstract code; the compilable C++ code. Note that the C++ code snippets include a scalar and vector part, which is automatically computed from the abstract code. In this case, a constant has been pulled out of the loop and named _lio_1.

The code generation pipeline in Brian is illustrated in Figure 2. Code generation will typically start with a set of (potentially stochastic) first order ordinary differential equations. Using an appropriate solver, these equations are converted into a sequence of update rules. As an example, consider the simple equation d*v*/dt = −*v*/τ mentioned above. Brian will detect that the equation is linear and can be solved exactly, and will therefore generate the following update rule: v_new = v_old * exp(-dt/tau).

Such strings or sequences of strings form a sort of mathematical pseudocode called an *abstract code block*. The user can also specify abstract code blocks directly. For example, to define the operation that is executed upon a spike, the user might write 'v_post += w' as shown above.

From an abstract code block, Brian transforms the statements into one of a number of different target languages. The simplest is to generate Python code, using NumPy for vectorized operations. This involves relatively few transformations of the abstract code, mostly concerned with indexing. For example, for a reset operation *v*←*v*_*r*_ that should be carried out only on those neurons that have spiked, code equivalent to v[has_spiked] = v_r is generated, where has_spiked is an array of integers with the indices of the neurons that have spiked. The direct C++ code generation target involves a few more transformations on the original code, but is still relatively straightforward. For example, the power operation *a*^*b*^ is written as a**b in Python, whereas in C++ it should be written as pow(a, b). This is implemented using Python's built-in AST module, which transforms a string in Python syntax into an abstract syntax tree that can be iterated. Finally, there is the Cython code generation target. Cython is a Python package that allows users to write code in a Python-like syntax and have it automatically converted into C++, compiled and run. This allows Python users to maintain easy-to-read code that does not have the performance limitations of pure Python.

The result of these transformations is a block of code in a different target language called a *snippet*, because it is not yet a complete *compilable source file*. This final transformation is carried out by the widely used Python templating engine Jinja2, which inserts the snippet into a template file.

The final step is the compilation and execution of the source files. Brian operates in one of two main modes: *runtime* or *standalone* mode. The default runtime mode is managed directly by Python. Source files are compiled into separate Python modules which are then imported and executed in sequence by Brian. This allows users to mix arbitrary pure Python code with compiled code, but comes with a performance cost, namely that each function call has an associated Python overhead. For large numbers of neurons this difference is relatively little because the majority of time is spent inside compiled code rather than in Python overheads (which are a fixed cost not depending on the number of neurons). However, for smaller networks that might need to be run repeatedly or for a long duration, these overheads can be significant. Brian therefore also has the standalone mode, in which it generates a complete C++ project that can be compiled and run entirely independently of Python and Brian. This is transparent for the users and only requires them to write set_device('cpp_standalone') at the beginning of their scripts. While this mode comes with the advantage of increased performance and portability, it also implies some limitations as user-specified Python code and generated code cannot be interspersed.

Brian's code generation framework has been designed in a modular fashion and can be extended on multiple levels. For specific models, the user might want to integrate a simulation with hand-written code in the target programming language, e.g., to feed real-time input from a sensor into the simulation. Brian supports this use case by allowing references to arbitrary user-defined functions in the model equations and statements, if its definition in the target language and the physical dimensions of its arguments and result are provided by the user. On a global level, Brian supports the definition of new target languages and devices. This mechanism has for example been used to provide GPU functionality through the *Brian2GeNN* interface (Nowotny et al., 2014; Stimberg et al., 2014–2018b), generating and executing model code for the GeNN simulator (Yavuz et al., 2016).

#### 2.1.4. Numerical integration

As stated above, Brian converts differential equations into a sequence of statements that integrate the equations numerically over a single time step. If the user does not choose a specific integration method, Brian selects one automatically. For linear equations, it will solve the equations exactly according to their analytic solution. In all other cases, it will chose a numerical method, using an appropriate scheme for stochastic differential equations if necessary. The exact methods that will be used by this default mechanism depend on the type of the model. For single-compartment neuron and synapse models, the methods *exact, euler*, and *heun* (see explanation below) will be tried in order, and the first suitable method will be applied. Multicompartmental neuron models will chose from the methods *exact, exponential euler, rk2*, and *heun*.

The following integration algorithms are provided by Brian and can be chosen by the user:

- *exact* (named *linear* in previous versions): exact integration for linear equations
- *exponential euler*: exponential Euler integration for conditionally linear equations
- *euler*: forward Euler integration (for additive stochastic differential equations using the Euler-Maruyama method)
- *rk2*: second order Runge-Kutta method (midpoint method)
- *rk4*: classical Runge-Kutta method (RK4)
- *heun*: stochastic Heun method for solving Stratonovich stochastic differential equations with non-diagonal multiplicative noise.
- *milstein*: derivative-free Milstein method for solving stochastic differential equations with diagonal multiplicative noise

In addition to these predefined solvers, Brian also offers a simple syntax for defining new solvers (for details see Stimberg et al., 2014).

#### 2.1.5. Data and execution model

In terms of data and execution, a Brian simulation is essentially just an ordered sequence of code blocks, each of which can modify the values of variables, either scalars or vectors (of fixed or dynamic size). For example, *N* neurons with the same equations are collected in a NeuronGroup object. Each variable of the model has an associated array of length *N*. A code block will typically consist of a loop over indices *i* = 0, 1, 2, …, *N*−1 and be defined by a block of code executing in a *namespace* (a dictionary mapping names to values). Multiple code objects can have overlapping namespaces. So for example, for neurons there will be one code object to perform numerical integration, another to check threshold crossing, another to perform post-spike reset, etc. This adds a further layer of flexibility, because the user can choose to re-order these operations, for example to choose whether synaptic propagation should be carried out before or after post-spike reset.

Each user defined variable has an associated index variable that can depend on the iteration variable in different ways. For example, the numerical integration iterates over *i* = 0, 1, 2, …, *N*−1. However, post-spike reset only iterates over the indices of neurons that spiked. Synapses are handled in the same way. Each synapse has an associated presynaptic neuron index, postsynaptic neuron index, and synaptic index and the resulting code will be equivalent to v_post[postsynaptic_index[i]] += w[synaptic_index[i]].

Brian assumes an unrestricted memory model in which all variables are accessible, which gives a particularly flexible scheme that makes it simple to implement many non-standard models. This flexibility can be achieved for medium scale simulations running on a single CPU (the most common use case of Brian). However, especially for synapses, this assumption may not be compatible with all code generation targets where memory access is more restrictive (e.g., in MPI or GPU setups). As a consequence, not all models that can be defined and run in standard CPU targets will be able to run efficiently in other target platforms.

### 2.2. GeNN

GeNN (GPU enhanced Neuronal Networks) (Nowotny, 2011; Knight et al., 2012-2018; Yavuz et al., 2016) is a C++ and NVIDIA CUDA (Wikipedia, 2006; NVIDIA Corporation, 2006-2017) based framework for facilitating neuronal network simulations with GPU accelerators. It was developed because optimizing simulation code for efficient execution on GPUs is a difficult problem that distracts computational neuroscience researchers from focusing on their core research. GeNN uses code generation to achieve efficient GPU code while maintaining maximal flexibility of what is being simulated and which hardware platform to target.

#### 2.2.1. Main modeling focus

The focus of GeNN is on spiking neuronal networks. There are no restrictions or preferences for neuron model and synapse types, albeit analog synapses such as graded synapses and gap junctions do affect the speed performance strongly negatively.

**Figure:**
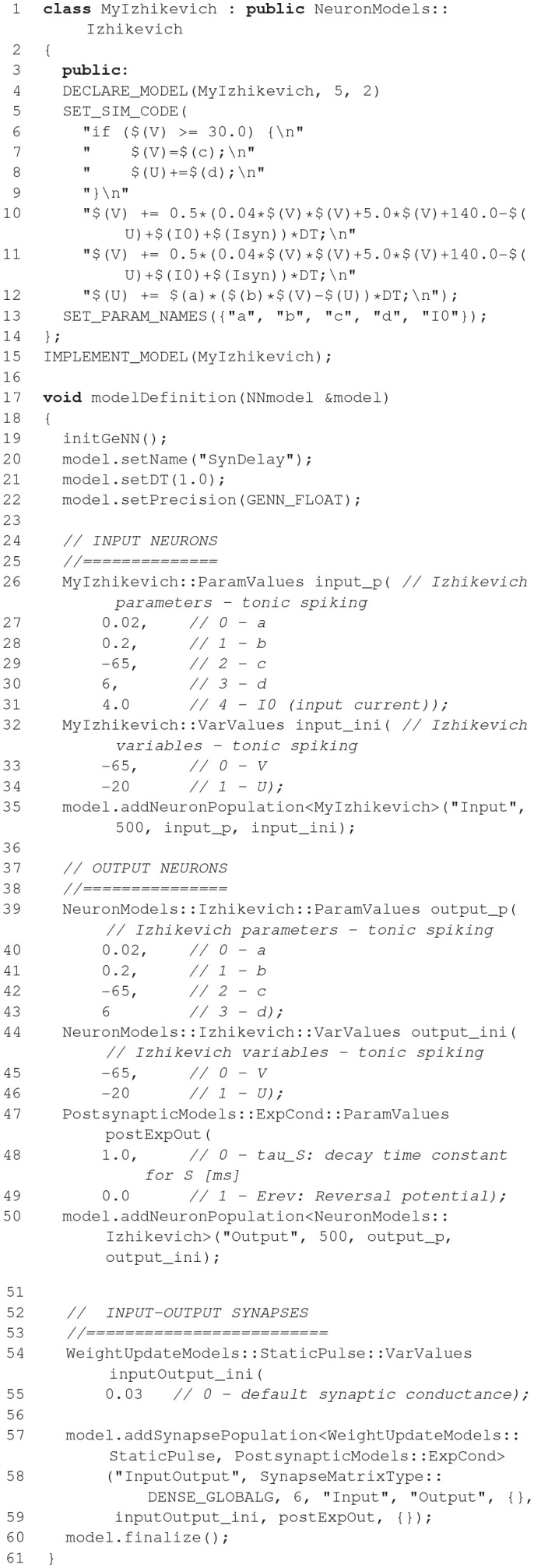


The code example above illustrates the nature of the GeNN API. GeNN expects users to define their own code for neuron and synapse model time step updates as C++ strings. In the example above, the neurons are standard Izhikevich neurons and synaptic connections are pulse coupling with delay. GeNN works with the concept of neuron and synapse types and subsequent definition of neuron and synapse populations of these types.

#### 2.2.2. Code generation pipeline

**Figure 3:**
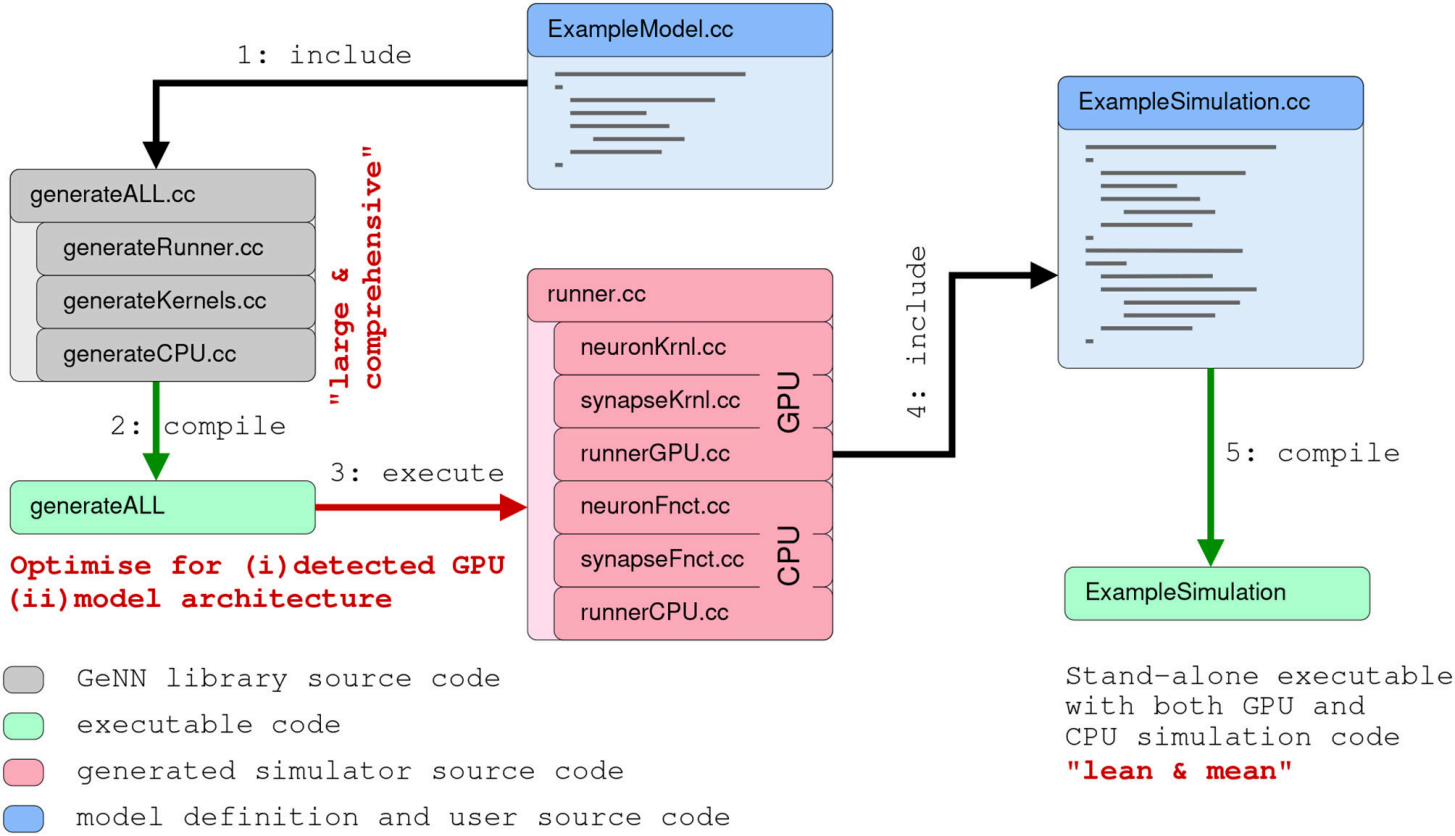
Schematic of the code generation flow for the GPU simulator framework GeNN. Neural models are described in a C/C++ model definition function (“ExampleModel.cc”), either hand-crafted by a user or generated from a higher-level model description language such as SpineML or Brian 2 (see main text). The neuron model description is included into the GeNN compiler that produces optimized CUDA/C++ code for simulating the specified model. The generated code can then be used by hand-crafted or independently generated user code to form the final executable. The framework is minimalistic in generating only optimized CUDA/C++ code for the core model and not the simulation workflow in order to allow maximal flexibility in the deployment of the final executable. This can include exploratory or large scale simulations but also real-time execution on embedded systems for robotics applications. User code in blue, GeNN components in gray, generated CUDA/C++ code in pink.

The model description provided by the user is used to generate C++ and CUDA C code for efficient simulation on GPU accelerators. For maximal flexibility, GeNN only generates the code that is specific to GPU acceleration and accepts C/C++ user code for all other aspects of a simulation, even though a number of examples of such code is available to copy and modify. The basic strategy of this workflow is illustrated in Figure 3. Structuring the simulator framwork in this way allows achieving key goals of code generation in the GPU context. First, the arrangement of neuron and synapse populations into kernel blocks and grids can be optimized by the simulator depending on the model and the hardware detected at compile time. This can lead to essential improvements in the simulation speed. The approach also allows users and developers to define a practically unlimited number of neuron and synapse models, while the final, generated code only contains what is being used and the resulting executable code is lean. Lastly, accepting the users' own code for the input-output and simulation control allows easy integration with many different usage scenarios, ranging from large scale simulations to using interfaces to other simulation tools and standards and to embedded use, e.g., in robotics applications.

#### 2.2.3. Numerical integration

Unlike for other simulators, the numerical integration methods, and any other time-step based update methods are for GeNN in the user domain. Users define the code that performs the time step update when defining the neuron and synapse models. If they wish to use a numerical integration method for an ODE based neuron model, users need to provide the code for their method within the update code. This allows for maximal flexibility and transparency of the numerical model updates.

However, not all users may wish to use the C++ interface of GeNN or undertake the work of implementing the time step updates for their neuron models from scratch. For these users there are additional tools that allow connecting other model APIs to GeNN. Brian2GeNN (Nowotny et al., 2014; Stimberg et al., 2014–2018b) allows to execute Brian 2 (see section 2.1 Stimberg et al., 2014) scripts with GeNN as the backend and there is a separate toolchain connecting SpineCreator and SpineML (see section 2.8; Richmond et al., 2014) to GeNN to achieve the same. Although there can be a loss in computing speed and the range of model features that can be supported when using such interfaces, using GPU acceleration through Brian2GeNN can be as simple as issuing the command set_device('genn') in a Python script for Brian 2.

### 2.3. Myriad

The goal of the Myriad simulator project (Rittner and Cleland, 2014) is to enable the automatic parallelization and multiprocessing of any compartmental model, particularly those exhibiting dense analog interactions such as graded synapses and mass diffusion that cannot easily be parallelized using standard approaches. This is accomplished computationally via a shared-memory architecture that eschews message-passing, coupled with a radically granular design approach that flattens hierarchically defined cellular models and can subdivide individual isometric compartments by state variable. Programmatically, end-user models are defined in a Python-based environment and converted into fully-specified C99 code (for CPU or GPU) via code generation techniques that are enhanced by a custom abstract syntax tree (AST) translator and, for NVIDIA GPUs, a custom object specification for CUDA enabling fully on-card execution.

#### 2.3.1. Main modeling focus

Myriad was conceived as a strategy to enable the parallelization of densely integrated mechanisms in compartmental models. Under traditional message-passing approaches to parallelization, compartment states that update one another densely–e.g., at every timestep—cannot be effectively parallelized. However, such dense analog interactions are common in compartmental models; examples include graded synapses, gap junctions, and charge or mass diffusion among adjacent compartments. In lieu of message passing, Myriad uses a shared memory strategy with barrier synchronization that parallelizes dense models as effectively as sparsely coupled models. This strategy imposes scale limitations on simulations based on available memory, though these limitations are being somewhat eased by new hardware developments.

#### 2.3.2. Model notation

The core of Myriad is a *parallel solver layer* designed so that all models that can be represented as a list of isometric, stateful nodes (compartments), can be connected pairwise by any number of arbitrary mechanisms and executed with a high degree of parallelism on CPU threads. No hierarchical relationships among nodes are recognized during execution; hierarchies that exist in user-defined models are flattened during code generation. This flat organization facilitates thread-scaling to any number of available threads and load-balancing with very fine granularity to maximize the utilization of available CPU or GPU cores. Importantly, analog coupling mechanisms such as cable equations, Hodgkin-Huxley membrane channels, mass diffusion, graded synapses, and gap junctions can be parallelized in Myriad just as efficiently as sparse events. Because of this, common hierarchical relationships in neuronal models, such as the positions of compartments along an extended dendritic tree, can be flattened and the elements distributed arbitrarily across different compute units. For example, two nodes representing adjacent compartments are coupled by “adjacency” mechanisms that pass appropriate quantities of charge and mass between them without any explicit or implicit hierarchical relationship. This solver comprises the lowest layer of a three-layer simulator architecture.

A top-level *application layer*, written in idiomatic Python 3 enriched with additional C code, defines the object properties and primitives available for end-user model development. It is used to specify high-level abstractions for neurons, sections, synapses, and network properties. The mechanisms (particles, ions, channels, pumps, etc.) are user-definable with object-based inheritance, e.g., channels inherit properties based on their permeant ions. Simulations are represented as objects to facilitate iterative parameter searches and reproducibility of results. The inheritance functionality via Python's native object system allows access to properties of parent component and functionality can be extended and overridden at will.

The intermediate *interface layer* flattens and translates the model into non-hierarchical nodes and coupling mechanisms for the solver using AST-to-AST translation of Python code to C. Accordingly, the top-level model definition syntax depends only on application-layer Python modules; in principle, additional such modules can be written for applications outside neuroscience, or to mimic the model definition syntax of other Python-based simulators. For the intended primary application of solving dense compartmental models of neurons and networks, the models are defined in terms of their cellular morphologies and passive properties (e.g., lengths, diameters, cytoplasmic resistivity) and their internal, transmembrane, and synaptic mechanisms. State variables include potentials, conductances, and (optionally) mass species concentrations. Equations for mechanisms are arbitrary and user-definable.

#### 2.3.3. Code generation pipeline

To achieve an efficient parallelization of dense analog mechanisms, it was necessary to eschew message-passing. Under message-based parallelization, each data transfer between compute units generates a message with an uncertain arrival time, such that increased message densities dramatically increase the rollback rate of speculative execution and quickly become rate-limiting for simulations. Graded connections such as analog synapses or cable equations yield new messages at every timestep and hence parallelize poorly. This problem is generally addressed by maintaining coupled analog mechanisms on single compute units, with parallelization being limited to model elements that can be coupled via sparse boolean events, such as action potentials (Hines and Carnevale, 2004). Efficient simulations therefore require a careful, platform-specific balance between neuronal complexity and synaptic density (Migliore et al., 2006). The unfortunate consequence is that platform limitations drive model design.

In lieu of message passing, Myriad is based on a uniform memory access (UMA) architecture. Specifically, every mechanism reads all parameters of interest from shared memory, and writes its output to shared memory, at every fixed timestep. Shared memory access, and a global clock that regulates barrier synchronization among all compute units (thereby coordinating all timesteps), are GPU hardware features. For parallel CPU simulations, the OpenMP 3.1+ API for shared-memory multiprocessing has implicit barrier and reduction intrinsics that provide equivalent, platform-independent functionality. Importantly, while this shared-memory design enables analog interactions to be parallelized efficiently, to take proper advantage of this capacity on GPUs, the simulation must execute on the GPU independently rather than being continuously controlled by the host system. To accomplish this, Myriad uses a code generation strategy embedded in its three-layer architecture (see section 2.3.2). The lowest (solver) layer is written in C99 for both CPUs and NVIDIA GPUs (CUDA). The solver requires as input a list of isometric nodes and a list of coupling mechanisms that connect pairs of nodes, all with fully explicit parameters defined prior to compilation (i.e., execution of a Myriad model requires just-in-time compilation of the solver). To facilitate code reuse and inheritance from the higher (Python) layers, a custom-designed minimal object framework implemented in C (Schreiner, 1999) supports on-device virtual functions; to our knowledge this is the first of its kind to execute on CUDA GPUs. The second, or interface, layer is written in Python; this layer defines top-level objects, instantiates the node and mechanism dichotomy, converts the Python objects defined at the top level into the two fully-specified lists that are passed to the solver, and manages communication with the simulation binaries. The top, or application layer, will comprise an expandable library of application-specific modules, also written in Python. These modules specify the relevant implementations of Myriad objects in terms familiar to the end user. For neuronal modeling, this could include neurite lengths, diameters, and branching, permeant ions (mass and charge), distributed mechanisms (e.g., membrane channels), point processes (e.g., synapses), and cable equations, among other concepts common to compartmental simulators. Additional top-layer modules can be written by end users for different purposes, or to support different code syntaxes.

Execution of a Myriad simulation begins with a transformation of the user-specified model definition into two Python lists of node and mechanism objects. Parameters are resolved, and the Python object lists are transferred to the solver layer via a custom-built Python-to-C pseudo-compiler (*pycast*; an AST-to-AST translator from Python's native abstract syntax tree (AST) to the AST of *pycparser* (a Myriad dependency), facilitated by Myriad's custom C object framework). These objects are thereby rendered into fully explicit C structs which are compiled as part of the simulation executable. The choice of CPU or GPU computation is specified at execution time via a compiler option. On CPUs and compliant GPUs, simulations execute using dynamic parallelism to maximize core utilization (via OpenMP 3.1+ for CPUs or CUDA 5.0+ on compute capability 3.5+ GPUs).

The limitation of Myriad's UMA strategy is scalability. Indeed, at its conception, Myriad was planned as a simulator on the intermediate scale between single neuron and large network simulations because its shared-memory, barrier synchronization-dependent architecture limited the scale of simulations to those that could fit within the memory of a single high-speed chassis (e.g., up to the memory capacity of a single motherboard or CUDA GPU card). However, current and projected hardware developments leveraging NVIDIA's NVLink interconnection bus (NVIDIA Corporation, 2014) are likely to ease this limitation.

#### 2.3.4. Numerical integration

For development purposes, Myriad supports the fourth-order Runge-Kutta method (RK4) and the backward Euler method. These and other methods will be benchmarked for speed, memory requirements, and stability prior to release.

### 2.4. NESTML

NESTML (Plotnikov et al., 2016; Blundell et al., 2018; Perun et al., 2018a) is a relatively new modeling language, which currently only targets the NEST simulator (Gewaltig and Diesmann, 2007). It was developed to address the maintainability issues that followed from a rising number of models and model variants and ease the model development for neuroscientists without a strong background in computer science. NESTML is available unter the terms of the GNU General Public License v2.0 on GitHub (https://github.com/nest/nestml; Perun et al., 2018b) and can serve as a well-defined and stable target platform for the generation of code from other model description languages such as NineML (Raikov et al., 2011) and NeuroML (Gleeson et al., 2010).

#### 2.4.1. Main modeling focus

The current focus of NESTML is on integrate-and-fire neuron models described by a number of differential equations with the possibility to support compartmental neurons, synapse models, and also other targets in the future.

**Figure:**
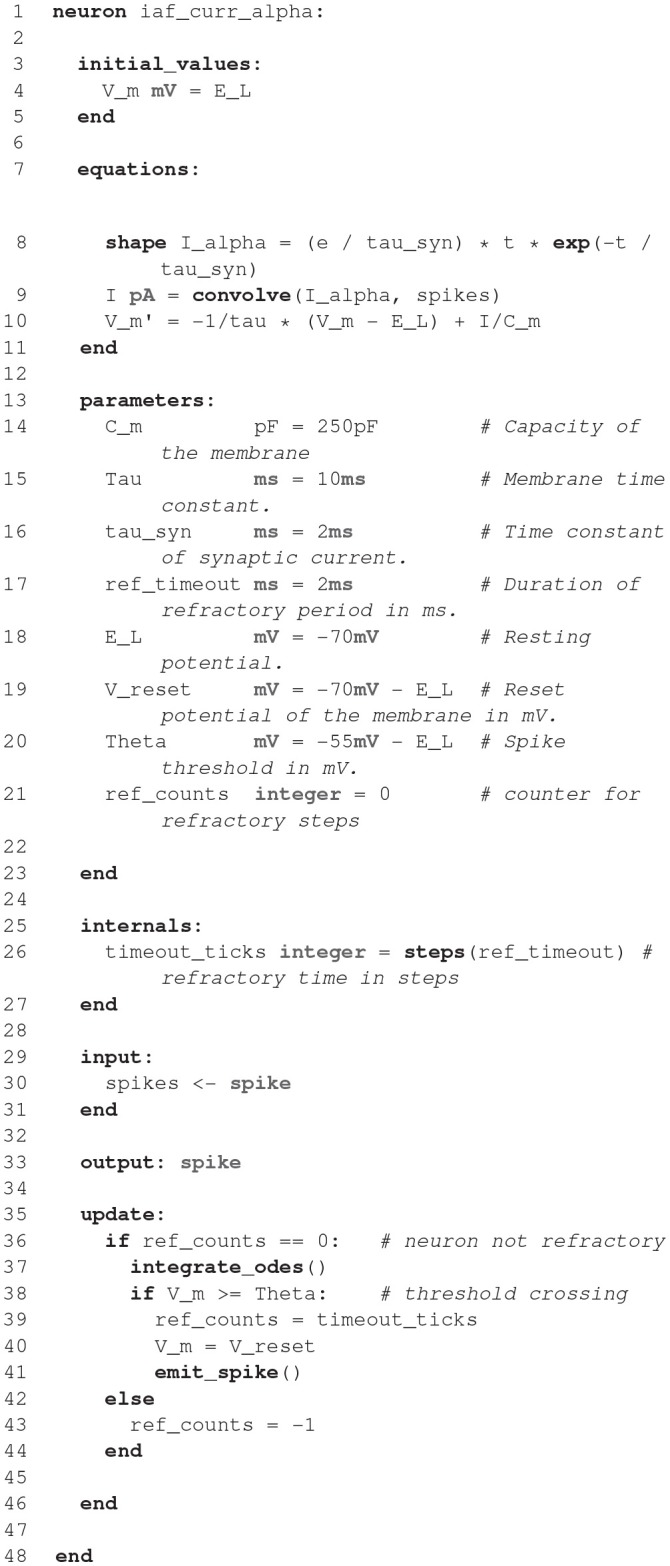


The code shown in the listing above demonstrates the key features of NESTML with the help of a simple current-based integrate-and-fire neuron with alpha-shaped synaptic input as described in section 1. A neuron in NESTML is composed of multiple blocks. The whole model is contained in a *neuron* block, which can have three different blocks for defining model variables: *initial_values, parameters*, and *internals*. Variable declarations are composed of a non-empty list of variable names followed by their type. Optionally, initialization expressions can be used to set default values. The type can either be a plain data type such as *integer* and *real*, a physical unit (e.g., *mV*) or a composite physical unit (e.g., *nS/ms*).

Differential equations in the *equations* block can be used to describe the time evolution of variables in the *initial_values* block. Postsynaptic shapes and synonyms inside the *equations* block can be used to increase the expressiveness of the specification.

The type of incoming and outgoing events are defined in the *input* and *output* blocks. The neuron dynamics are specified inside the *update* block. This block contains an implementation of the propagation step and uses a simple embedded procedural language based on Python.

#### 2.4.2. Code generation pipeline

In order to have full freedom for the design, the language is implemented as an external domain specific language (DSL; van Deursen et al., 2000) with a syntax similar to that of Python. In contrast to an internal DSL an external DSL doesn't depend syntactically on a given host language, which allows a completely customized implementation of the syntax and results in a design that is tailored to the application domain.

Usually external DSLs require the manual implementation of the language and its processing tools. In order to avoid this task, the development of NESTML is backed by the language workbench MontiCore (Krahn et al., 2010). MontiCore uses context-free grammars (Aho et al., 2006) in order to define the abstract and concrete syntax of a DSL. Based on this grammar, MontiCore creates classes for the abstract syntax (*metamodel*) of the DSL and parsers to read the model description files and instantiate the metamodel.

NESTML is composed of several specialized sublanguages. These are composed through language embedding and a language inheritance mechanism: *UnitsDSL* provides all data types and physical units, *ExpressionsDSL* defines the style of Python compatible expressions and takes care of semantic checks for type correctness of expressions, *EquationsDSL* provides all means to define differential equations and postsynaptic shapes and *ProceduralDSL* enables users to specify parts of the model in the form of ordinary program code. In situations where a modeling intent cannot be expressed through language constructs this allows a more fine-grained control than a purely declarative description could.

The decomposition of NESTML into sublanguages enables an agile and modular development of the DSL and its processing infrastructure and independent testing of the sublanguages, which speeds up the development of the language itself. Through the language composition capabilities of the MontiCore workbench the sublanguages are composed into the unified DSL NESTML.

NESTML neurons are stored in simple text files. These are read by a parser, which instantiates a corresponding abstract syntax tree (AST). The AST is an instance of the metamodel and stores the essence of the model in a form which is easily processed by a computer. It completely abstracts the details of the user-visible model representation in the form of its concrete syntax. The symbol table and the AST together provide a semantic model.

**Figure 4:**
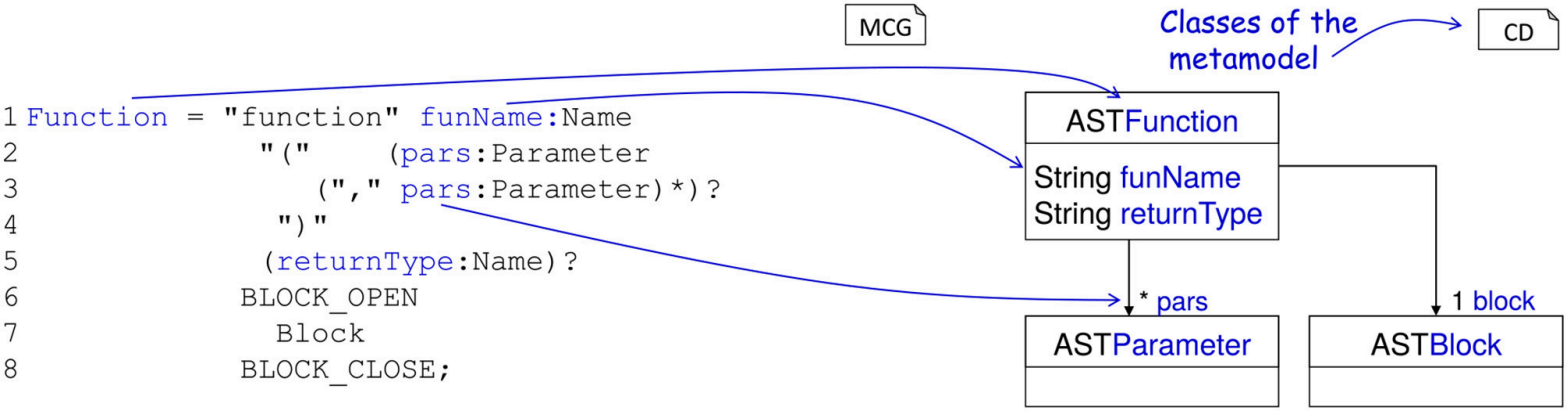
Example definition of a NESTML concept and generation of the AST. **(Left)** A production for a function in NESTML. The lefthandside defines the name of the production, the righthandside defines the production using terminals, other productions and special operators (*, ?). A function starts with the keyword function followed by the function's name and an optional list of parameters enclosed in parentheses followed by the optional return value. Optional parts are marked with ?. The function body is specified by the production (Block) between two keywords. **(Right)** The corresponding automatically derived meta-model as a class diagram. Every production is mapped to an AST class, which is used in the further language processing steps.

Figure 4 shows an excerpt of the NESTML grammar and explains the derivation of the metamodel. A grammar is composed of a non-empty set of *productions*. For every production a corresponding class in the metamodel is created. Based on the right hand side of the productions attributes are added to this class. Classes can be specified by means of specifications of explicit names in the production names of attributes in the metamodel.

**Figure 5:**
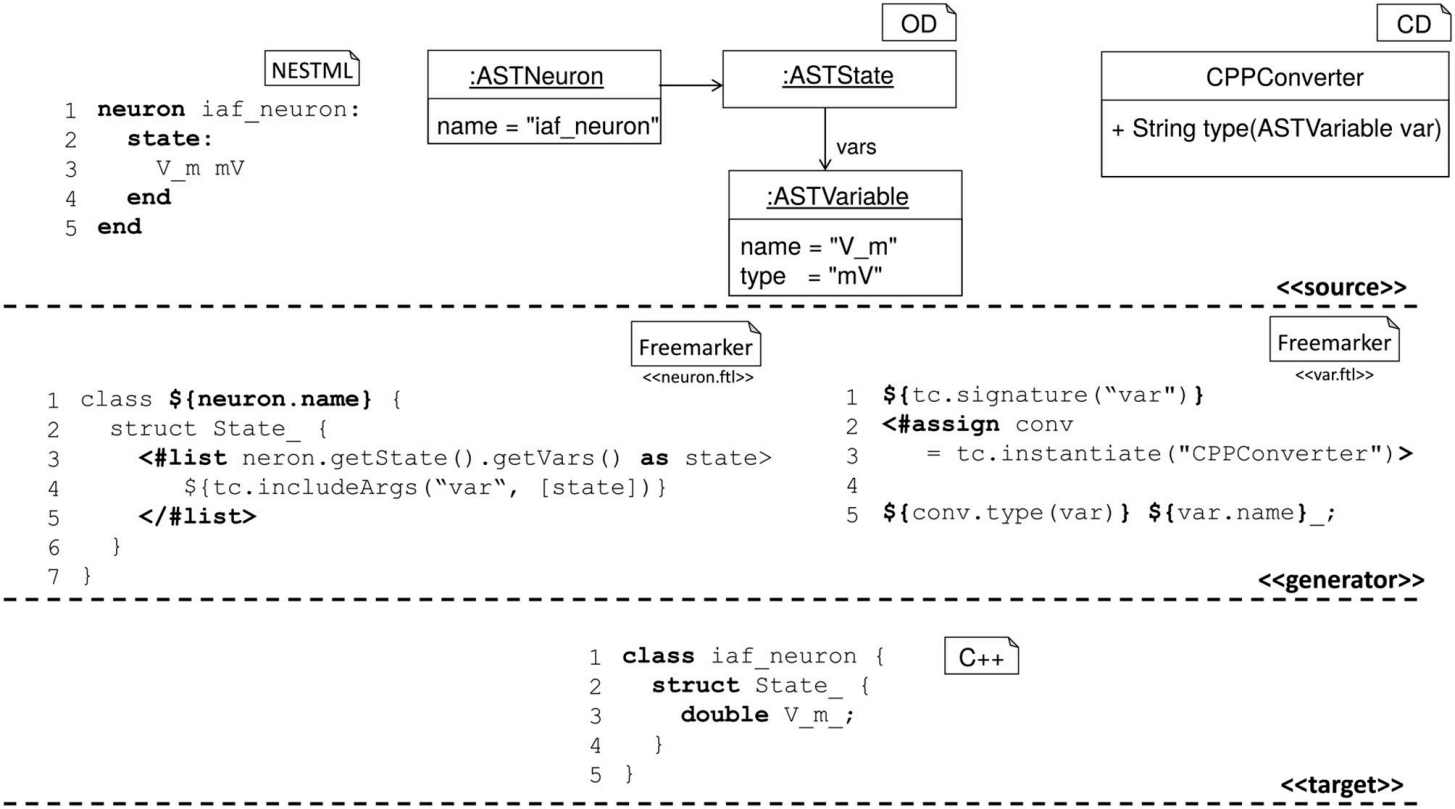
Components for the code generation in NESTML. **(Top)** Source model, corresponding AST, and helper classes. **(Middle)** Templates for the generation of C++ code. The left template creates a C++ class body with an embedded C++ struct, the right template maps variable name and variable type using a helper class. The template on the left includes the template on the right once for each state variable defined in the source model. **(Bottom)** A C++ implementation as created from the source model using the generation templates.

NEST expects a model in the form of C++ code, using an internal programming interface providing hooks for parameter handling, recording of state variables, receiving and sending events, and updating instances of the model to the next simulation time step. The NESTML model thus needs to be transformed to this format (Figure 5).

For generating the C++ code for NEST, NESTML uses the code generation facilities provided by the MontiCore workbench, which are based on the template engine Freemarker (https://freemarker.apache.org/). This approach enables a tight coupling of the model AST and the symbol table, from which the code is generated, with the text based templates for the generation of code.

Before the actual code generation phase, the AST undergoes several model to model transformations. First, equations and shapes are extracted from the NESTML AST and passed to an analysis framework based on the symbolic math package SymPy (Meurer et al., 2017). This framework (Blundell et al., 2018) analyses all equations and shapes and either generates explicit code for the update step or code that can be handled by a solver from the GNU Scientific Library (https://gnu.org/software/gsl/). The output of the analysis framework is a set of model fragments which can again be instantiated as NESTML ASTs and integrated into the AST of the original neuron and replace the original equations and shapes they were generated from.

Before writing the C++ code, a *constant folding* optimization is performed, which uses the fact that internal variables in NESTML models do not change during the simulation. Thus, expressions involving only internal variables and constants can be factored out into dedicated expressions, which are computed only once in order to speed up the execution of the model.

#### 2.4.3. Numerical integration

NESTML differentiates between different types of ODEs. ODEs are categorized according to certain criteria and then assigned appropriate solvers. ODEs are solved either analytically if they are linear constant coefficient ODEs and are otherwise classified as stiff or non stiff and then assigned either an implicit or an explicit numeric integration scheme.

### 2.5. Neuroml/LEMS

*NeuroML* version 1 (*NeuroML1* henceforth; Goddard et al., 2001; Gleeson et al., 2010) was originally conceived as a simulator-agnostic domain specific language (DSL) for building biophysically inspired models of neuronal networks, focusing on separating model description from numerical implementation. As such, it provided a fixed set of components at three broad layers of abstraction: morphological, ion channel, and network, which allowed a number of pre-existing models to be described in a standardized, structured format (Gleeson et al., 2010). The role of code generation in *NeuroML1* pipelines was clear—the agnostic, abstract model definition needed to be eventually mapped into concrete implementations (e.g., code for NEURON; Carnevale and Hines, 2006; GENESIS; Bower and Beeman, 1998) in order for the models to be simulated.

Nevertheless, the need for greater flexibility and extensibility beyond a predefined set of components and, more importantly, a demand for lower level model descriptions also described in a standardized format (contrarily to *NeuroML1*, where for example component dynamics were defined textually in the language reference, thus inaccessible from code) culminated in a major language redesign (referred to as *NeuroML2*), underpinned by a second, lower level language called *Low Entropy Model Specification* (*LEMS*; Cannon et al., 2014).

#### 2.5.1. Main modeling focus

*LEMS* can be thought of as a meta-language for defining domain specific languages for networks (in the sense of graphs), where each node can have local dynamics described by ordinary differential equations, plus discrete state jumps or changes in dynamical regimes mediated by state-dependent events—also known as *Hybrid Systems* (van der Schaft and Schumacher, 2000). *NeuroML2* is thus a DSL (in the computational neuroscience domain) defined using *LEMS*, and as such provides standardized, structured descriptions of model dynamics up to the ODE level.

#### 2.5.2. Model notation

**Figure 6:**
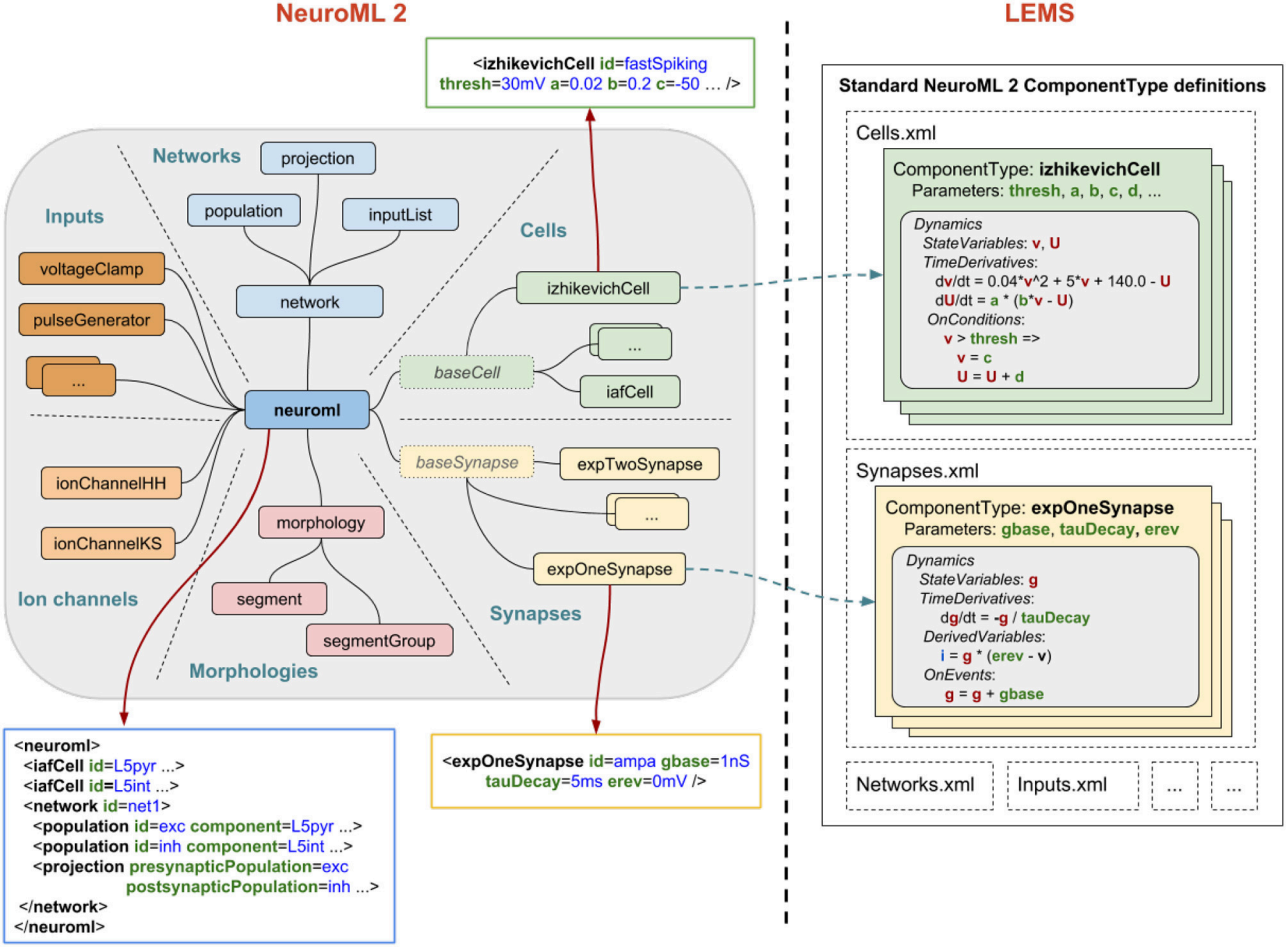
NeuroML2 and LEMS. *NeuroML2* is a language which defines a hierarchical set of elements used in computational models in neuroscience in the following broad categories: *Networks, Cells, Synapses, Morphologies, Ion Channels*, and *Inputs*. These provide the building blocks for specifying 3D populations of cells, both morphologically detailed and abstract, connected via a variety of (plastic) chemical and electrical synapses receiving external spike or current based stimuli. Examples are shown of the (truncated) XML representations of: (blue) a network containing two populations of integrate-and-fire cells connected by a single projection between them; (green) a spiking neuron model as described by Izhikevich (2003); (yellow) a conductance based synapse with a single exponential decay waveform. On the right the definition of the structure and dynamics of these elements in the *LEMS* language is shown. Each element has a corresponding *ComponentType* definition, describing the parameters (as well as their dimensions, not shown) and the dynamics in terms of the state variables, the time derivative of these, any derived variables, and the behavior when certain conditions are met or (spiking) events are received. The standard set of *ComponentType* definitions for the core *NeuroML2* elements are contained in a curated set of files (*Cells.xml, Synapses.xml*, etc.) though users are free to define their own *ComponentTypes* to extend the scope of the language.

An overview of *NeuroML2* and *LEMS* is depicted in Figure 6, illustrating how *Components* for an abstract cell model (*izhikevichCell*) and a synapse model (*expOneSynapse*) can be specified in XML (i.e., in the computational neuroscience domain, only setting required parameters for the *Components*), with the definitions for their underlying models specified in *LEMS ComponentTypes* which incorporate a description of the dimensions of the parameters, the dynamical state variables and behavior when certain conditions or events occur.

Besides providing more structured information describing a given model and further validation tools for building new ones (Cannon et al., 2014), *NeuroML2*-*LEMS* models can be directly parsed, validated, and simulated via the *jLEMS* interpreter (Cannon et al., 2018), developed in Java.

#### 2.5.3. Code generation pipeline

Being derived from *LEMS*, a metalanguage designed to generate simulator-agnostic domain-specific languages, *NeuroML2* is prone to be semantically different at varying degrees from potential code generation targets. As discussed elsewhere in the present article (sections 2.1 and 2.4), code generation boils down to trivial template merging or string interpolation once the source and target models sit at comparable levels of abstraction (reduced “impedance mismatch”), implying that a number of semantic processing steps might be required in order to transform *LEMS*/*NeuroML2* into each new target. Given *LEMS*/*NeuroML2*'s low-level agnosticism—there is always the possibility that it will be used to generate code for a yet-to-be-invented simulator—*NeuroML2* infrastructure needs to be flexible enough to adapt to different strategies and pipelines.

**Figure 7:**
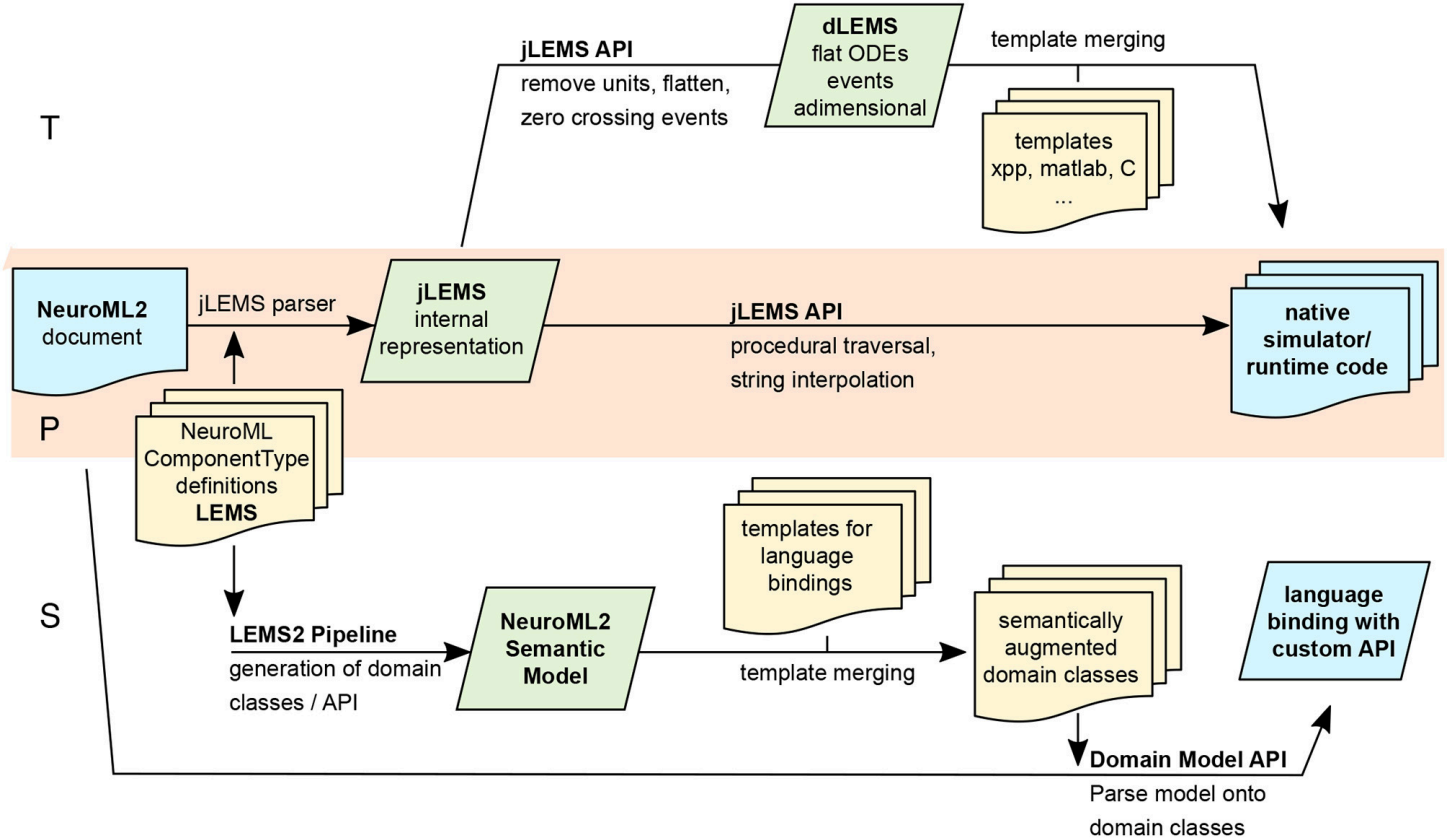
Multiple pipelines involving code generation for *NeuroML2* and *LEMS*. Purely Procedural (**P**) and intermediate representation/Template-based (**T**) pipelines, both stemming from the internal representation constructed by *jLEMS* from parsed *LEMS XML* documents. **S**: Generation of customizable language bindings via construction of *LEMS* Semantic model and merging with templates.

This flexibility is illustrated in Figure 7, where *NeuroML2* pipelines involving code generation are outlined. Three main strategies are discussed in detail: a procedural pipeline starting from *jLEMS*'s internal structures (Figure 7**P**), which as the first one to be developed, is the most widely tested and supports more targets; a pipeline based on building an intermediate representation semantically similar to that of typical neuronal modeling / hybrid-system-centric numerical software, which can then be merged with templates (as decoupled as possible from *LEMS* internals) for each target format (Figure 7**T**); and a customizable language binding generator, based on an experimental compiler infrastructure for *LEMS* which provides a rich semantic model with validation and automatic generation of traversers (Figure 7**S**)—akin to semantic models built by language workbenches such as MontiCore, which has been employed to build NESTML (section 2.4).

##### 2.5.3.1. jLEMS runtime and procedural generation

The *jLEMS* simulator was built alongside the development of the *LEMS* language, providing a testbed for language constructs and, as such, enables parsing, validating, and interpreting of *LEMS* documents (models). *LEMS* is canonically serialized as *XML*, and the majority of existing models have been directly developed using this syntax. In order to simulate the model, *jLEMS* builds an internal representation conforming to *LEMS* semantics (Cannon et al., 2014). This loading of the *LEMS XML* into this internal state is depicted as a green box in the **P** (middle) branch of Figure 7. Given that any neuronal or general-purpose simulator will eventually require similar information about the model in order to simulate it, the natural first approach to code generation from *LEMS* involved procedural interaction with this internal representation, manually navigating through component hierarchies to ultimately fetch dynamics definitions in terms of *Parameters, DerivedVariables*, and routing events. Exporters from *NeuroML2* to *NEURON* (both hoc and mod), *Brian1* and *SBML* were developed using these techniques (end point of Figure 7 **P**), and can be found in the org.neuroml.export repository (Gleeson et al., 2018).

Even if all the information required to generate code for different targets is encoded in the *jLEMS* intermediate representation, the fact that the latter was designed to support a numerical simulation engine creates overheads for the procedural pipeline, typically involving careful mixed use of *LEMS* / domain abstractions and requiring repetitive application of similar traversal/conversion patterns for every new code generator. This regularity suggested pursuing a second intermediate representation, which would capture these patterns into a further abstraction.

##### 2.5.3.2. Lower-level intermediate representation/templating

Neuronal simulation engines such as *Brian, GENESIS, NEST* and *NEURON* tend to operate at levels of abstraction suited to models described in terms of differential equations (e.g., explicit syntax for time derivatives in *Brian, NESTML* and *NEURON* nmodl), in conjunction with discontinuous state changes (usually abstracted within “event handlers” in neuronal simulators). Code generation for any of those platforms from *LEMS* model would thus be facilitated if *LEMS* models could be cast at this level of abstraction, as most of the transformations would consist of one-to-one mappings which are particularly suited for template-based generation. Not surprisingly, *Component* dynamics in *LEMS* are described precisely at the hybrid dynamical system level, motivating the construction of a pipeline (Figure 7 **T**) centered around an intermediate representation, termed *dLEMS* (Marin et al., 2018b), which would facilitate simplified code generation not only for neuronal simulators (*dLEMS* being semantically close to e.g., *Brian* and *NESTML*) but also for ODE-aware general purpose numerical platforms like *Matlab* or even *C/Sundials* (Hindmarsh et al., 2005).

Besides reducing development time by removing complex logic from template bodies—all processing is done on the semantic model, using a general purpose language (*Java* in the case of *jLEMS* backed pipelines) instead of the templating DSL, which also promotes code reuse—this approach also enables target language experts to work with templates with reduced syntactic noise, shifting focus from processing information on *LEMS* internals to optimized generation (e.g., more idiomatic, efficient code).

##### 2.5.3.3. Syntax oriented generation/semantic model construction

Both the procedural and template-based pipelines (Figure 7 **P**, **T**) described in the preceding paragraphs stem from the *jLEMS internal representation* data structure, which is built from both the *LEMS* document and an implementation of *LEMS* semantics, internal to *jLEMS*. To illustrate the interplay between syntax and semantics, consider for example the concept of *ComponentType extension* in *LEMS*, whereby a *ComponentType* can inherit structure from another. In a *LEMS* document serialized as *XML*, the “child” *ComponentType* is represented by an XML element, with an attribute (string) containing the name of the “parent.” Syntactically, there is no way of determining that this string should actually represent an existing *ComponentType*, and that structure should be inherited—that is the role of semantic analysis.

The **P** and **T** pipelines rely heavily on APIs for traversing, searching, and transforming a semantic model. They have been implemented on top of the one implemented by *jLEMS*—even though it contains further transformations introduced to ease interpretation of models for numerical simulation—the original purpose of *jLEMS*. Given that both code generation and interpretation pipelines depend on the initial steps of parsing the concrete syntax (XML) and building a semantic model with novel APIs, a third “semantic” pipeline (Figure 7 **S**) is under development to factor out commonalities. Starting with *LEMS* definitions for a domain-specific language—in the particular case of *NeuroML2*, a collection of *ComponentType*s spanning the domain of biophysical neuronal models—a semantic model is produced in the form of domain types for the target language, via template-based code generation. Any (domain specific, e.g., *NeuroML2*) *LEMS* document can then be unmarshalled into domain objects, constituting language bindings with custom APIs that can be further processed for code generation or used in an interpreter.

Any *LEMS*-backed language definition (library of *ComponentType*s) can use the experimental *Java* binding generator directly through a *Maven* plugin we have created (Marin and Gleeson, 2018). A sample project where domain classes for *NeuroML2* are built is available (Marin et al., 2018a), illustrating how to use the plugin.

##### 2.5.3.4. Numerical integration

As a declarative model specification language, *LEMS* was designed to separate model description from numerical implementation. When building a model using *LEMS*—or any DSL built on top of it such as *NeuroML2*—the user basically instantiates preexisting (or creates new and then instantiates) *LEMS ComponentTypes*, parameterizing and connecting them together hierarchically. In order to simulate this model, it can either be interpreted by the native *LEMS* interpreters (e.g., *jLEMS*, which employs either Forward-Euler or a 4th order Runge-Kutta scheme to approximate solutions for ODE-based node dynamics and then performs event detection and propagation) or transform the models to either general-purpose languages or domain-specific simulators, as described above for each code generation pipeline.

#### 2.5.4. General considerations and future plans

Different code generation strategies for *LEMS* based domain languages —such as *NeuroML2*—have been illustrated. With *LEMS* being domain and numerical implementation agnostic, it is convenient to continue with complementary approaches to code generation, each one fitting different users' requirements. The first strategy to be developed, fully procedural generation based on *jLEMS* internal representation (**P**), has lead to the most complex and widely tested generators to date—such as the one from *NeuroML2* to *NEURON* (mod/hoc). Given that *jLEMS* was not built to be a high-performance simulator, but a reference interpreter compliant with *LEMS* semantics, it is paramount to have robust generation for state-of-the art domain-specific simulators if *LEMS*-based languages are to be more widely adopted. Conversely, it is important to lower the barriers for simulator developers to adopt *LEMS*-based models as input. These considerations have motivated building the *dLEMS*/templating based code generation pipeline (**T**), bringing *LEMS* abstractions into a representation closer to that of most hybrid-system backed solvers, so that simulator developers can relate to templates resembling the native format, with minimal interaction with *LEMS* internals.

The semantic-model/custom API strategy (**S**) is currently at an experimental stage, and was originally designed to factor out parsing/semantic analysis from *jLEMS* into a generic compiler front end-like (Grune et al., 2012) standalone package. This approach was advantageous in comparison with the previous XML-centric strategy, where bindings were generated from XML Schema Descriptions manually built and kept up-to-date with *LEMS ComponentType* definitions—which incurred in redundancy as *ComponentTypes* fully specify the structure of a domain document (*Component* definitions). While it is experimental, the modular character of this new infrastructure should contribute to faster, more reusable development of code generators for new targets.

### 2.6. NineML, Pype9, 9ML-toolkit

The Network Interchange for NEuroscience Modeling Language (NineML) (Raikov et al., 2011) was developed by the International Neuroinformatics Coordinating Facility (INCF) NineML taskforce (2008–2012) to promote model sharing and reusability by providing a mathematically-explicit, simulator-independent language to describe networks of point neurons. Although the INCF taskforce ended before NineML was fully specified, the component-based descriptions of neuronal dynamics designed by the taskforce informed the development of both LEMS (section 2.5; Cannon et al., 2014) and SpineML (section 2.8; Richmond et al., 2014), before the NineML Committee (http://nineml.net/committee) completed version 1 of the specification in 2015 (https://nineml-spec.readthedocs.io/en/1.1).

NineML only describes the model itself, not solver-specific details, and is therefore suitable for exchanging models between a wide range of simulators and tools. One of the main aims of the NineML Committee is to encourage the development of an eco-system of interoperable simulators, analysis packages, and user interfaces. To this end, the NineML Python Library (https://nineml-python.readthedocs.io) has been developed to provide convenient methods to validate, analyse, and manipulate NineML models in Python, as well as handling serialization to and from multiple formats, including XML, JSON, YAML, and HDF5. At the time of publication, there are two simulation packages that implement the NineML specification using code generation, PYthon PipelinEs for 9ml (Pype9; https://github.com/NeuralEnsemble/pype9) and the Chicken Scheme 9ML-toolkit (https://github.com/iraikov/9ML-toolkit), in addition to a toolkit for dynamic systems analysis that supports NineML through the NineML Python Library, PyDSTool (Clewley, 2012).

#### 2.6.1. Main modeling focus

The scope of NineML version 1 is limited to networks of point neurons connected by projections containing post-synaptic response and plasticity dynamics. However, version 2 will introduce syntax to combine dynamic components (support for “multi-component” dynamics components, including their flattening to canonical dynamics components, is already implemented in the NineML Python Library), allowing neuron models to be constructed from combinations of distinct ion channel and concentration models, that in principle could be used to describe models with a small number of compartments. Explicit support for biophysically detailed models, including large multi-compartmental models, is planned to be included in future NineML versions through a formal “extensions” framework.

#### 2.6.2. Model notation

NineML is described by an object model. Models can be written and exported in multiple formats, including XML, JSON, YAML, HDF5, Python, and Chicken Scheme. The language has two layers, the *Abstraction layer* (AL), for describing the behavior of network components (neurons, ion channels, synapses, etc.), and the *User layer*, for describing network structure. The AL represents models of hybrid dynamical systems using a state machine-like object model whose principle elements are *Regimes*, in which the behavior of the model state variables is governed by ordinary differential equations, and *Transitions*, triggered by conditions on state variable values or by external event signals, and which cause a change to a new regime, optionally accompanied by a discontinuous change in the values of state variables. For the example of a leaky integrate-and-fire model there are two regimes, one for the subthreshold behavior of the membrane potential, and one for the refractory period. The transition from subthreshold to refractory is triggered by the membrane potential crossing a threshold from below, and causes emission of a spike event and reset of the membrane potential; the reverse transition is triggered by the time since the spike passing a threshold (the refractory time period). This is expressed using YAML notation as follows:

**Figure:**
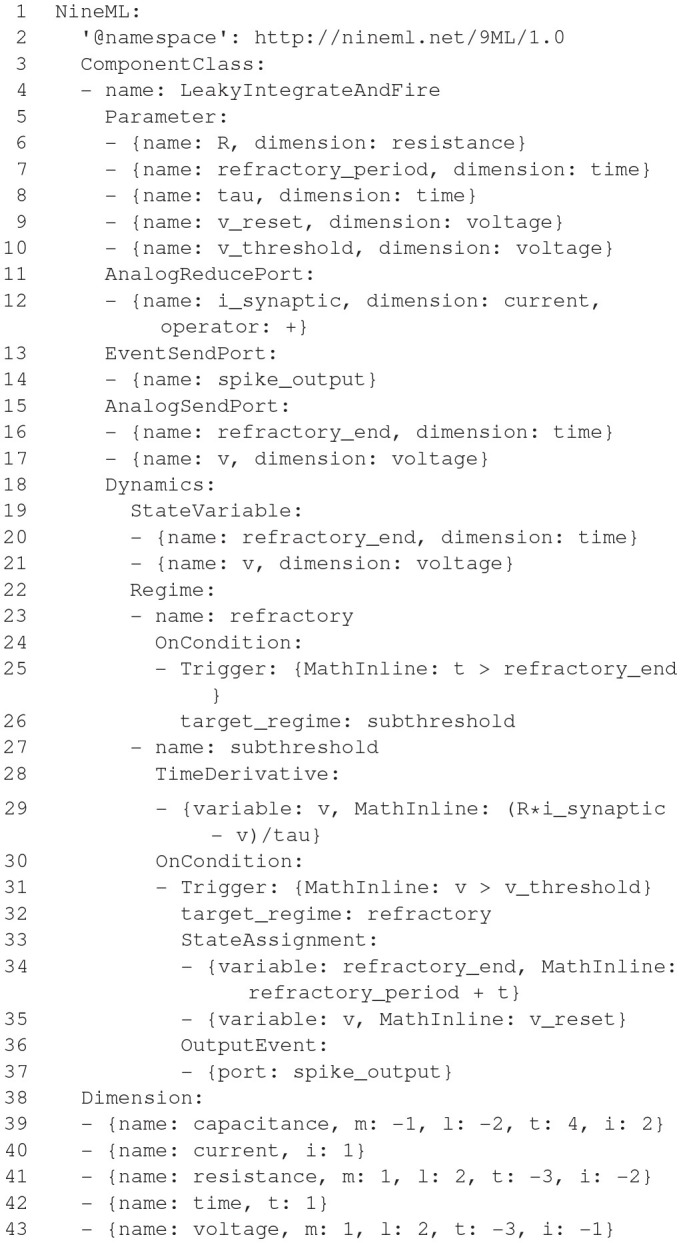


By design, the model description is intended to be a purely mathematical description of the model, with no information relating to the numerical solution of the equations. The appropriate methods for solving the equations are intended to be inferred by downstream simulation and code generation tools based on the model structure and their own heuristics. However, it is possible to add optional annotations to NineML models giving hints and suggestions for appropriate solver methods.

#### 2.6.3. Code generation pipelines

A number of tools have been developed to perform simulations from NineML descriptions.

**The NineML Python Library** (https://github.com/INCF/nineml-python) is a Python software library which maps the NineML object model onto Python classes, enabling NineML models to be expressed in Python syntax. The library also supports introspection, manipulation and validation of NineML model structure, making it a powerful tool for use in code generation pipelines. Finally, the library supports serialization of NineML models to and from multiple formats, including XML, JSON, YAML, and HDF5.

**Pype9** (https://github.com/NeuralEnsemble/pype9.git) is a collection of Python tools for performing simulations of NineML models using either NEURON or NEST. It uses the NineML Python library to analyze the model structure and manipulate it appropriately (for example merging linked components into a single component) for code generation using templating. Compilation of the generated code and linking with the simulator is performed behind the scenes.

**PyDSTool** (http://www2.gsu.edu/~matrhc/PyDSTool.htm) is an integrated environment for simulation and analysis of dynamical systems. It uses the NineML Python library to read NineML model descriptions, then maps the object model to corresponding PyDSTool model constructs. This is not code generation in any classical sense, although it could be regarded as generation of Python code. This is noted here to highlight the alternative ways in which declarative model descriptions can be used in simulation pipelines.

**9ML toolkit** (https://github.com/iraikov/9ML-toolkit) is a code generation toolkit for NineML models, written in Chicken Scheme. It supports the XML serialization of NineML as well as a NineML DSL based on Scheme. The toolkit generates executable code from NineML models, using native Runge-Kutta explicit solvers or the SUNDIALS solvers (Hindmarsh et al., 2005).

### 2.7. NEURON/NMODL

NEURON's (Hines and Carnevale, 1997) usefulness for research depends in large part on the ability of model authors to extend its domain by incorporating new biophysical mechanisms with a wide diversity of properties that include voltage and ligand gated channels, ionic accumulation and diffusion, and synapse models. At the user level these properties are typically most easily expressed in terms of algebraic and ordinary differential equations, kinetic schemes, and finite state machines. Working at this level helps the users to remain focused on the biology instead of low level programming details. At the same time, for reasonable performance, these model expressions need to be compiled into a variety of integrator and processor specific forms that can be efficiently integrated numerically. This functionality was made available in the NEURON Simulation Environment version 2 in 1989 with the introduction of the NEURON Model Description Language translator NMODL (Hines and Carnevale, 2000).

#### 2.7.1. Main modeling focus

NEURON is designed to model individual neurons and networks of neurons. It is especially suited for models where cable properties are important and membrane properties are complex. The modeling focus of NMODL is to desribe channels, ion accumulation, and synapses in a way that is independent of solution methods, threads, memory layout, and NEURON C interface details.

#### 2.7.2. Model notation

The example in Listing 1 shows how a voltage-gated current can be implemented and demonstrates the use of different language constructs. About 90 different constructs or keywords are defined in the NMODL language. Named blocks in NMODL have the general form of *KEYWORD { statements }*, and keywords are all upper case. The principle addition to the original MODL language was a NEURON block that specifies the name of the mechanism, which ions were used in the model, and which variables were functions of position on neuron trees. The *SUFFIX* keyword identifies this to be a density mechanism and directs all variable names declared by this mechanism to include the suffix *_kd* when referred to externally. This helps to avoid conflicts with similar names in other mechanisms. The mechanism has a *USEION* statement for each of the ions that it affects or is affected by. The *RANGE* keyword asserts that the specified variables are functions of position. In other words, each of these variables can have a different value in each neural compartment or segment.

The *UNITS* block defines new names for units in terms of existing names in the *UNIX* units database. The *PARAMETER* block declares variables whose values are normally specified by the user as parameters. The parameters generally remain constant during a simulation but can be changed. The *ASSIGNED* block is used for declaring two kinds of variables that are either given values outside the *mod* file or appear on the left hand side of assignment statements within the *mod* file. If a model involves differential equations, algebraic equations, or kinetic reaction schemes, their dependent variables or unknowns are listed in the *STATE* block. The *INITIAL* block contains instructions to initialize *STATE* variables. *BREAKPOINT* is a MODL legacy name (that perhaps should have been renamed to “CURRENT”) and serves to update current and conductance at each time step based on gating state and voltage values. The *SOLVE* statement tells how the values of the *STATE* variables will be integrated within each time step interval. NEURON has built-in routines to solve families of simultaneous algebraic equations or perform numeric integration which are discussed in section 2.7.4. At the end of a *BREAKPOINT* block all variables should be consistent with respect to time. The *DERIVATIVE* block is used to assign values to the derivatives of *STATE* variables described by differential equations. These statements are of the form *y*′ = *expr*, where a series of apostrophes can be used to signify higher-order derivatives. Functions are introduced with the *FUNCTION* keyword and can be called from other blocks like *BREAKPOINT, DERIVATIVE, INITIAL*, etc. They can be also called from the NEURON interpreter or other mechanisms by adding the suffix of the mechanism in which they are defined, e.g., *alpha_kd()*. One can enable or disable unit checking for specific code blocks using *UNITSON* or *UNITSOFF* keywords. The statements between *VERBATIM* and *ENDVERBATIM* will be copied to the translated C file without further processing. This can be useful for individual users as it allows addition of new features using the C language. But this should be done with great care because the translator program does not perform any checks for the specified statements in the *VERBATIM* block.

#### 2.7.3. Code generation pipeline

**Figure 8:**
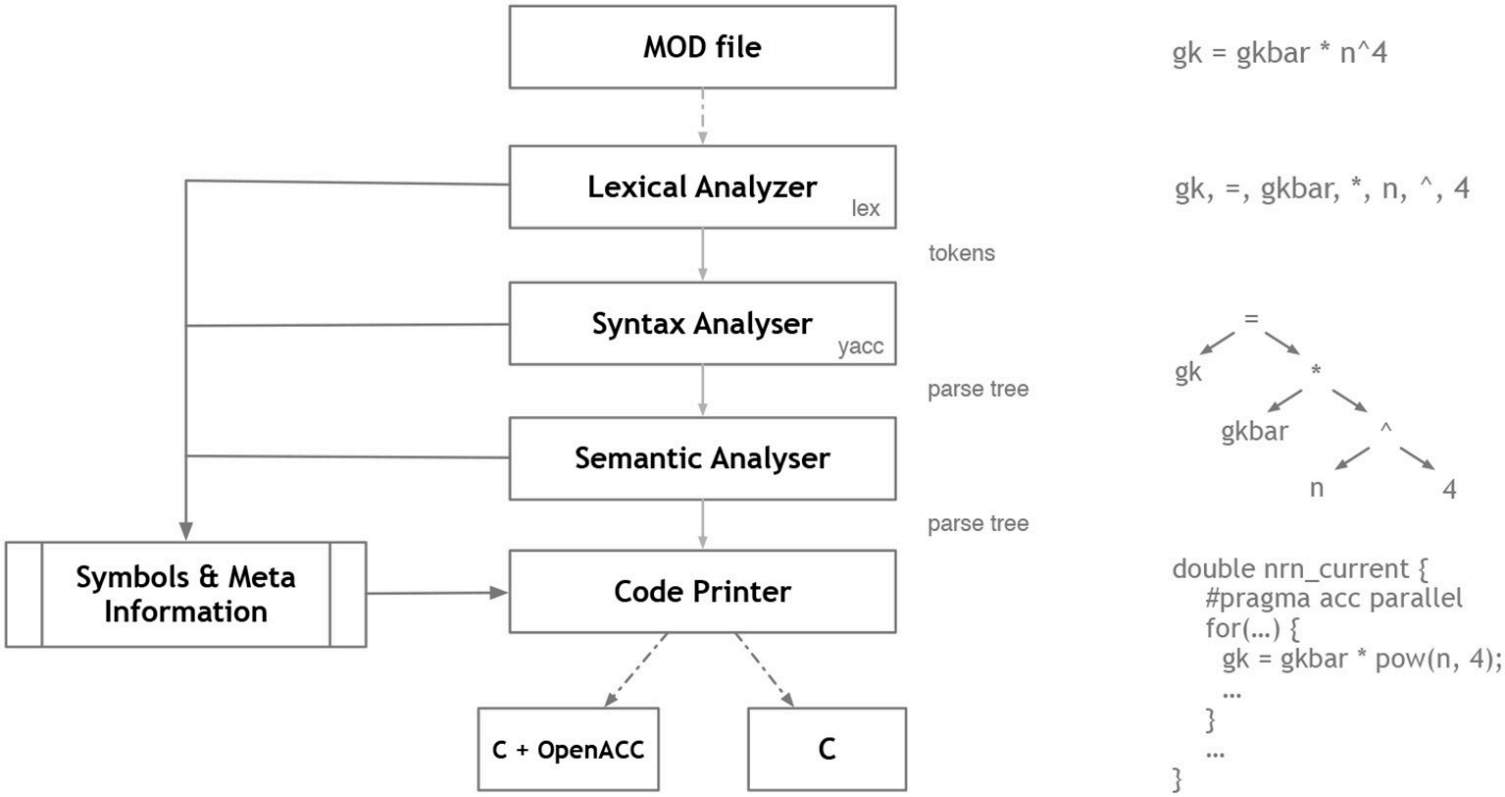
NMODL code generation workflow in NEURON/CoreNEURON targeting CPU/GPU.

NEURON has supported code generation with NMODL since version 2 released in 1989. Figure 8 shows the high level workflow of the source-to-source compiler that converts an NMODL description to a C file. The first step in this translation is lexical analysis which uses the lex/flex based lexical analyzer or scanner. The scanner reads the input NMODL file, recognizes lexical patterns in the source and returns tokens. These tokens are used by the next step called syntax analysis or parsing. The yacc/bison tool is used to generate the parser. Syntactic analysis is needed to determine if the series of tokens returned by the lexer are appropriate in a language—that is, whether or not the source statement has the right shape/form. For full syntactic analysis, the parser works with the lexer to generate a parse tree. However, not all syntactically valid sentences are meaningful and hence semantic analysis is performed. This analysis can catch errors like the use of undefined variables and incorrect uses of integration methods. During these steps, symbol tables are constructed and meta information about the model is stored in global data structures. This information is then used during the code printing step which writes C code to a file. These translation steps automatically handle details such as mass balance for each ionic species, different integration methods, units consistency, etc.

The output of the translator (a C file) is compiled and linked with the NEURON library to produce an executable. This achieves conceptual leverage and savings of effort not only because the high-level mechanism specification is much easier to understand and far more compact than the equivalent C code, but also because it spares the user from having to bother with low-level programming issues like how to “interface” the code with other mechanisms and with NEURON itself.

**Listing 1:**
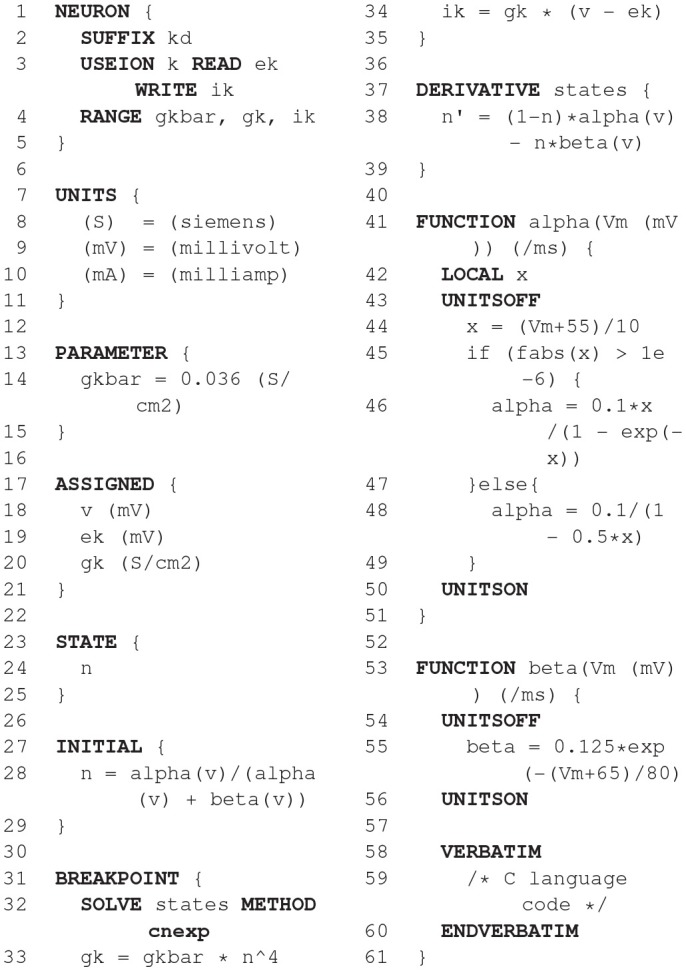
NMODL example of voltage-gated potassium current.

Over the years, the lexical analyzer and parser portions of the translator have been reasonably stable. The syntax extension needed to distinguish between density mechanisms and mechanisms localized to single points on a neuron, and the syntax extension needed to handle discrete spike event coupling to synapses, consisted of straightforward additions to the parser without any changes to the syntax. On the other hand, there have been a number of dramatic and far reaching changes in the processing of the parse tree and C code output as NEURON has evolved to make use of object oriented programming, variable step integrators (CVODE and IDA), threads, different memory layouts, and neural network simulations. In order to improve efficiency and portability on modern architectures like Intel Xeon Phi and NVIDIA GPUs, the core engine of the NEURON simulator is being factored out into the CoreNEURON simulator (Kumbhar et al., 2016). This simulator supports all NEURON models written in NMODL and uses a modified variant of the NMODL translator program called *mod2c*. This code generator supports memory layouts like Array-of-Structure (AoS) and *Structure-of-Array* (SoA) for efficient vectorization and memory access patterns. In order to support heterogeneous CPU/GPU platforms, mod2c generates code using the OpenACC programming model (Wikipedia, 2012).

#### 2.7.4. Numerical integration

The equations specified in the *DERIVATIVE* block are integrated using the numerical method specified by the *SOLVE* statement in the *BREAKPOINT* block. NEURON provides different methods for fixed step integration that include *cnexp, derivimplicit* which are appropriate for systems with widely varying time constants (stiff systems). The *cnexp* integration method is appropriate for mechanisms described by linear ODEs (including Hodgkin-Huxley-style channel models). This is an implicit integration method and can produce solutions that have second order precision in time. The *derivimplicit* integration method solves nonlinear ODEs and ODEs that include coupled state equations. This method provides first-order accuracy and is usable with general ODEs regardless of stiffness or non-linearity. If kinetic schemes are used, they get translated into equations and use the *sparse* solver, which produces results with first-order precision in time. It is important to note that independent of integration method selection, the high-level membrane description remains unchanged.

### 2.8. SpineML

The *Spiking Neural Mark-up Language* (SpineML) is a declarative XML based model description language for large scale neural network models (Richmond et al., 2014), based on the NineML syntax (see section 2.6; Raikov et al., 2011) and using the common model specification syntax of LEMS for components (section 2.5; Cannon et al., 2014). The declarative and simulator independent syntax of SpineML is designed to facilitate code generation to a number of simulation engines.

SpineML expands the NineML syntax, integrating new layers to support the ability to create and execute neural network experiments using a portable XML format. Primarily, two new layers have been added, a *Network* layer and an *Experiment* layer. These additions maximize the flexibility of described models, and provide an easy mapping for code-generation for complete networks.

**Figure 9:**
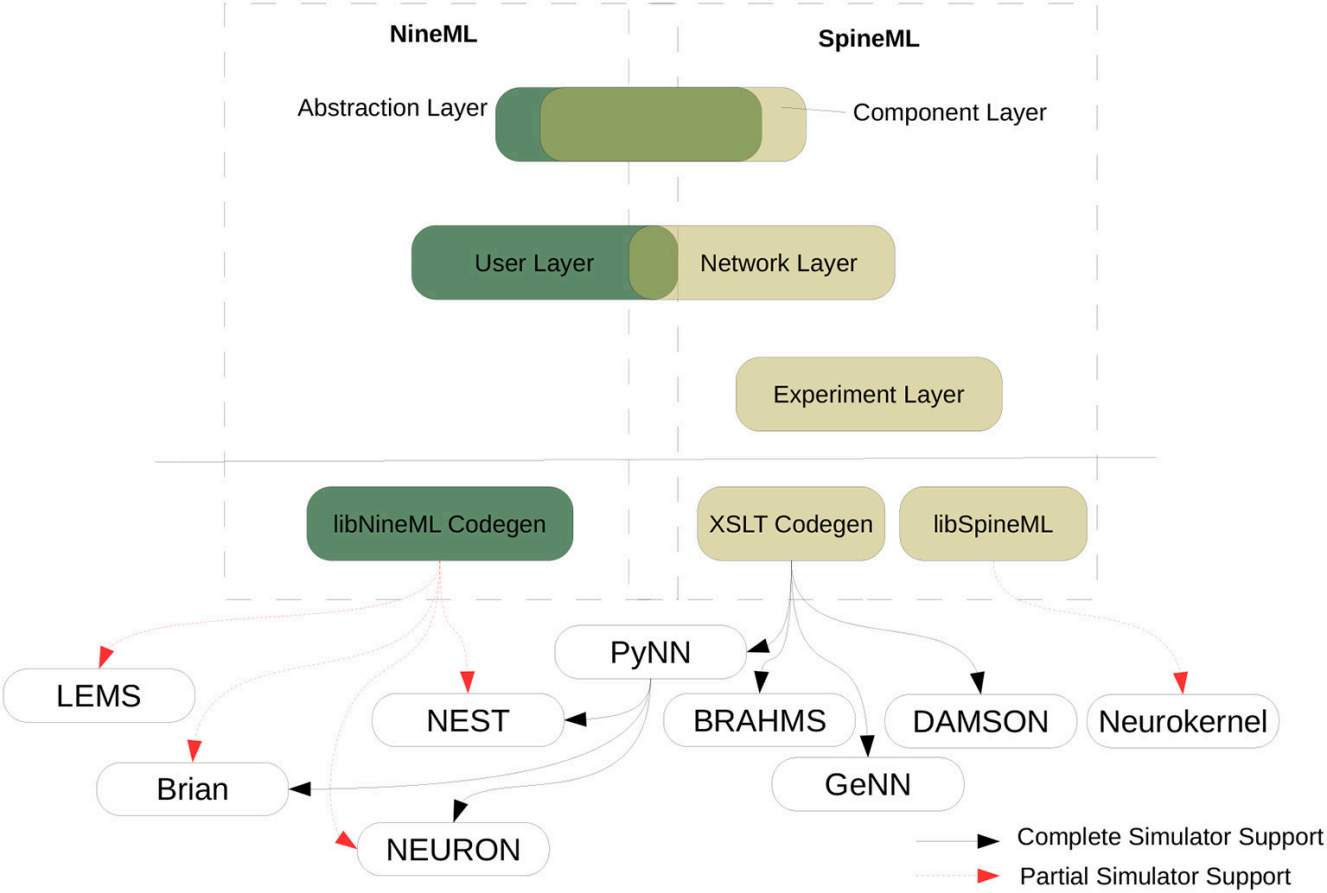
A comparison of the SpineML and NineML specification. The SpineML syntax is a proposed extension to the NineML modeling format which provides a complete syntax for describing models of spiking point neuron models with varying biological complexity. The SpineML syntax extends NineML and allows full simulator support for all three layers of components, networks and experiments (Adapted from Richmond et al., 2014).

Figure 9 details the structural overlap between the NineML and the SpineML formats. A three layer modeling approach is used to specify: components (e.g., neurons, synapses, etc.), a network connectivity pattern, and an experimental layer containing simulation specifications such as runtime conditions, population inputs and variable recording.

#### 2.8.1. Main modeling focus

**Figure 10:**
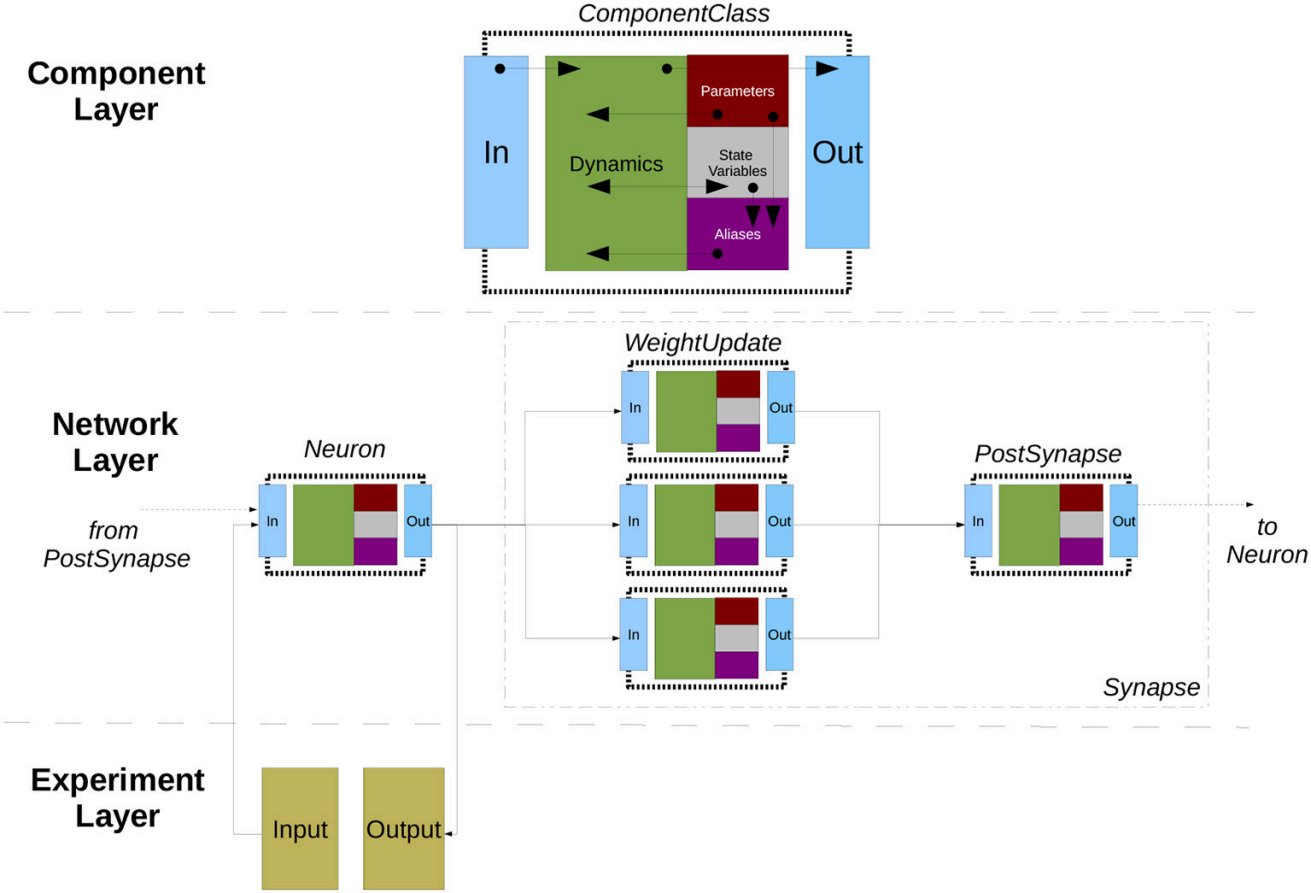
The modular dynamics within the three layers of SpineML. The figure shows the connectivity of a *Neuron* and *Synapse*, including *WeightUpdates* and a *PostSynapse* model. A *ComponentClass* described within the component layer defines the dynamical behavior of neurons, synapses, and neuromodulators. A *ComponentClass* updates state variables and emits outputs, by evolving differential equations, inputs, and aliases (parameters and state variables). *Input* and *Output* ports create an interface which enable each component instance to be connected to other instances within the network layer. The experiment layer defines network inputs such as spike sources or current injections (Taken from Richmond et al., 2014).

The syntax is designed primarily for the specification of large scale networks of point neurons but also has the flexibility to describe biologically constrained models consisting of non-standard components (such as gap junctions).The modeling focus is specifically designed around point neurons with arbitrary dynamics, expressed as any number of differential equations. Different behavioral regimes can be specified to allow expressive modeling of phenomena such as explicit refectory periods. As such, SpineML can represent much more complex neurons than Leaky Integrate and Fire, but is less well suited to multi-compartmental models such as Hodgkin-Huxley neurons. A SpineML project consists of three types of XML files: *component* files, the *network* file, and the *experiment* file (see Figure 10). Together these files describe a whole experiment, including component dynamics, network connectivity and experimental inputs and outputs.

#### 2.8.2. Model notation

**The Component Layer** encodes the individual computational modules (usually neuronal cells) of a simulation through the *ComponentClass* definition. The component level syntax of SpineML is directly derived from the NineML “abstraction” using LEMS, differing in two cases: the syntax for describing ports, and that SpineML units and dimensionality are combined into a single SI attribute.

**Listing 2:**
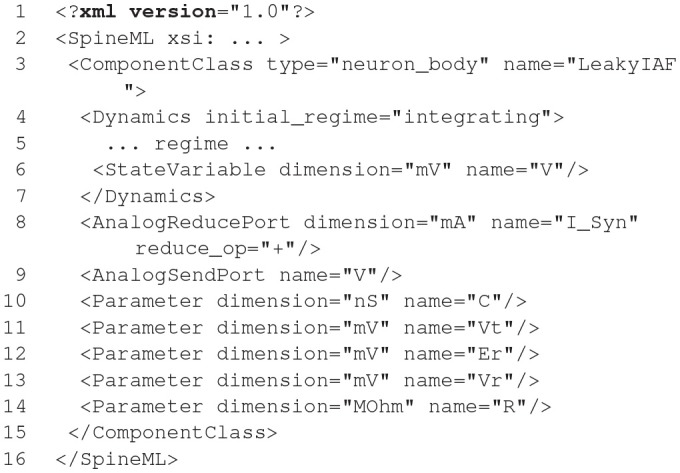
A SpineML Component representation of a leaky integrate-and-fire neuron. The definition of regimes has been moved to a separate listing.

SpineML components specify parameters, state variables, regimes, and ports. Parameters are static variables of the model which are referenced by time derivatives, state assignments and triggers. Along with state variables, parameters have both a name and a dimension consisting of an SI unit. Ports are defined to enable communication channels between components, and can be *Send* or *Receive* Ports. Ports are further divided onto *Analog* ports, for continuous variables, *Event* ports for events such as a spike, and *Impulse* ports for events with a magnitude. Listing 2 shows an example definition of a leaky integrate-and-fire component in SpineML. The component defines the *State Variable V, Parameters C, Vt, Er, Vr, R*, an output *AnalogueSendPort V* and an input *AnalogueReducePort I_Syn*.

The component defines the State-like “regimes” that change the underlying dynamics in response to events and changing conditions, as shown in Listing 3. A regime contains a time derivative, a differential equation that governs the evolution of a state variable. A regime can have transitions which change the current regime when a condition is met, that can further trigger events such as spiking outputs. State variables are referenced in the time derivatives, transitions, and conditions.

**Listing 3:**
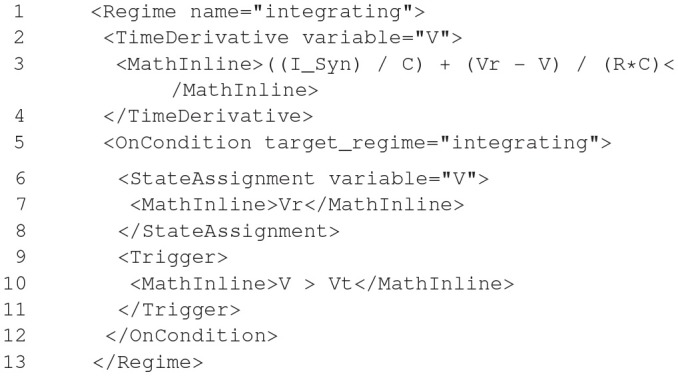
Integration regime for a leaky integrate-and-fire neuron.

**The Network Layer** description allows instances of components to be connected via ports using high level abstractions such as populations and projections. The complete object model of the network layer can be found in Richmond et al. (2014).

The high-level network syntax defines networks in terms of *Populations* and *Projections* defining *Synapse* components for *WeightUpdates* and *PostSynapse* primitives. A population can contain one or more *Projection*s to a named target *Population*, and each *Projection* can contain one or more *Synapses* which are associated with a connectivity pattern and sets of *WeightUpdate* and *PostSynapse* components.

A population property defines the instantiated state variable or parameter values of a named component. Property values can be described by a fixed value for all instances, statistical distributions, or as explicit value lists.

Population ports link the pre-synaptic and postsynaptic population, and can be *analog, event based*, or *impulse*. SpineML provides a special case, the *AnalogueReducePort*, which allows multiple postsynaptic values to be reduced using summation.

High-level abstractions of populations and projections simplify the descriptions of point-based network models allowing for a convenient mapping matching the abstraction of many simulators during code generation. However, projection based connectivity is not suitable for describing concepts such as gap junctions and neuromodulation. To address this the high-level object model has been extended to form an additional low-level schema. A low-level network allows the direct connection of components via *Inputs* and *Groups* of component instances. This provides a great deal of flexibility but requires simulators to support the connections of general computational components outside of the more common population projection abstraction level.

**The Experiment Layer** is the final phase of specifying a model and describes a simulation to be conducted. The syntax of the experimental layer is similar to the SED-ML experiment design language (Waltemath et al., 2011) but adds essential support for experiment inputs. It specifies the network model to be simulated, the period of simulation and the numerical integration scheme, the definition of model inputs, simulation inputs, and outputs.

#### 2.8.3. Code generation pipeline

A SpineML model can be mapped to a specific simulation engine using translation through code generation. Code generation for SpineML has been primarily provided through the use of XSLT templates. XSLT is an established method for document translation to HTML or other XML document formats. As there is no limit for the output file type generated from an XSLT template, it is suitable for any form of plain text file generation including native simulator source code generation. An XSLT processor works by recursively querying XML nodes using XPath expressions, and applying a template to process the content of each node. For simulator specific code, a model is processed by querying experiment, network layer, and component layer documents recursively using the branching and control elements of XSLT to generate plain text. As XSLT can be used as a fully functional programming language, it offers many advantages over a custom templating language, enabling complex control and data structures to inform the template output.

**Figure 11:**
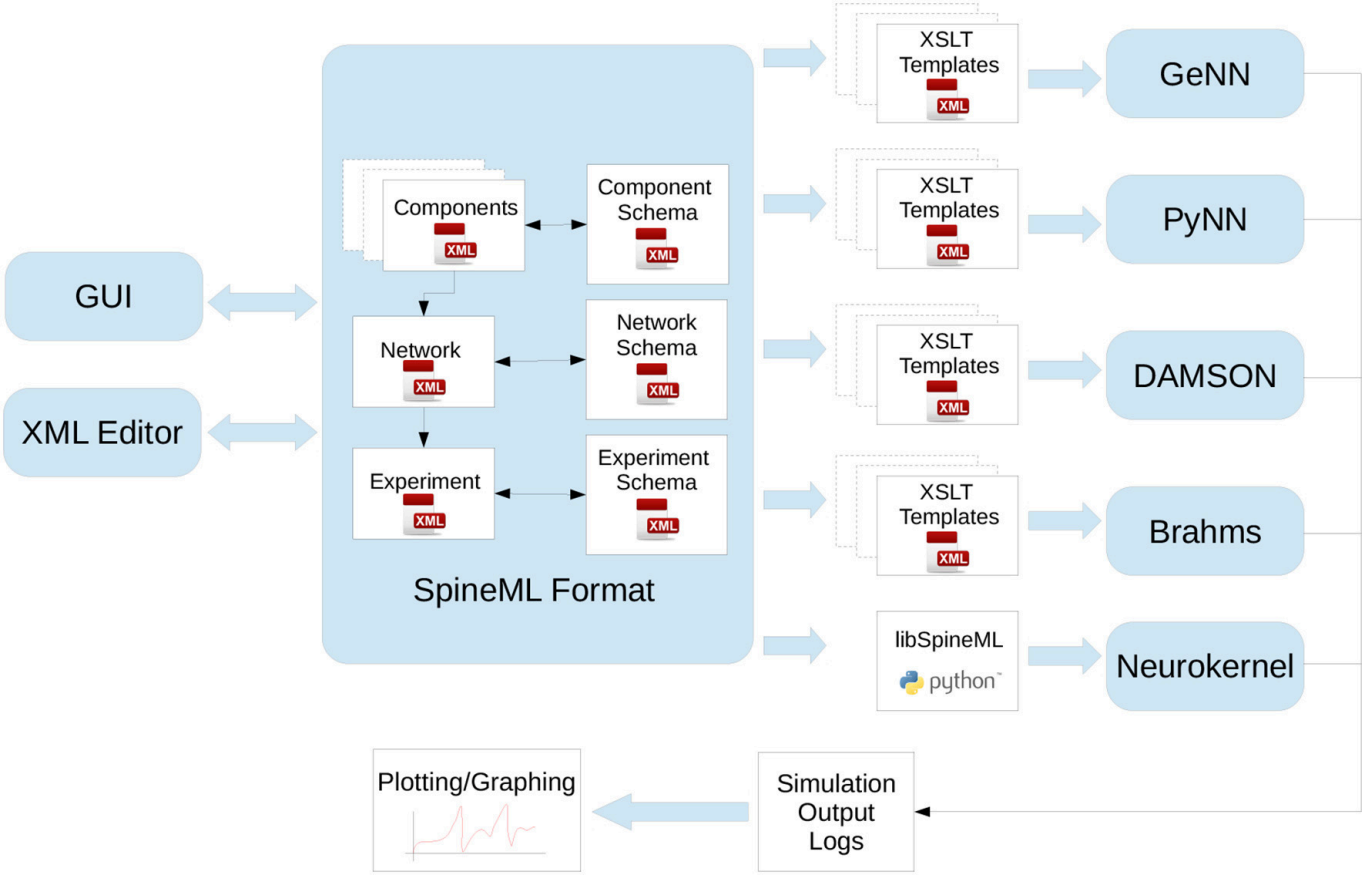
A tool-chain for simulation through code generation using the SpineML modeling syntax. The SpineML modeling syntax is composed of three layers, structured according to an XML Schema. Models can be generated manually using XML editors or using graphical user interface (GUI) tools. Translation of a model to any simulator is achieved by using a simulator specific set of XSLT templates, or Python libraries, to generate simulator code or native simulator model descriptions. Simulator code then logs results in a standardized format which can be used for plotting and analysis (Adapted from Richmond et al., 2014).

Code generation templates have been developed for a reference simulator, BRAHMS (Mitchinson et al., 2010): a multi-threaded simulation engine, DAMSON: a multi-processor multi-threaded event-driven form of C designed for emulating and compiling code for the SpiNNaker hardware architecture (Plana et al., 2007), GeNN: a GPU simulator for spiking neural systems (Yavuz et al., 2016), and a number of other simulators via PyNN (Figure 11).

Whilst SpineML models can be generated by hand, the use of a declarative common format allows independent tools to be generated for model design and creation using SpineML as a common storage format. Currently SpineCreator (Cope et al., 2017) provides a powerful GUI for SpineML generation with hooks into dynamic code generation and simulation output analysis.

Recently libSpineML has been released to add support for direct SpineML representation in Python, by deriving Python data structures from SpineML schema documents. This provides a convenient, programmatic wrapping to enable a new route for code generation from pythonic objects. Recent developments have demonstrated component level GPU code generation for the Neurokernel simulation platform (Givon and Lazar, 2016) using libSpineML and libSpineML2NK (Tomkins et al., 2016).

The libSpineML library enables SpineML objects to be imported, programmatically modified, and exported using a set of Python classes derived from the three SpineML layer schemata.

The libSpineML2NK library utilizes a general purpose SpineML-aware neuron model in the Neurokernel framework. By processing the libSpineML representation, the generic component model interfaces with the Neurokernel compute layer to dynamically allocate and manage GPU memory and manage network communication. Each SpineML component can then be converted to a NVIDIA CUDA kernel by translating the libSpineML object into a series of generic CUDA statements. Listing 3 shows an excerpt of a generated NVIDIA CUDA kernel, representing the integrating regime of a leaky integrate-and-fire SpineML component.

**Listing 4:**
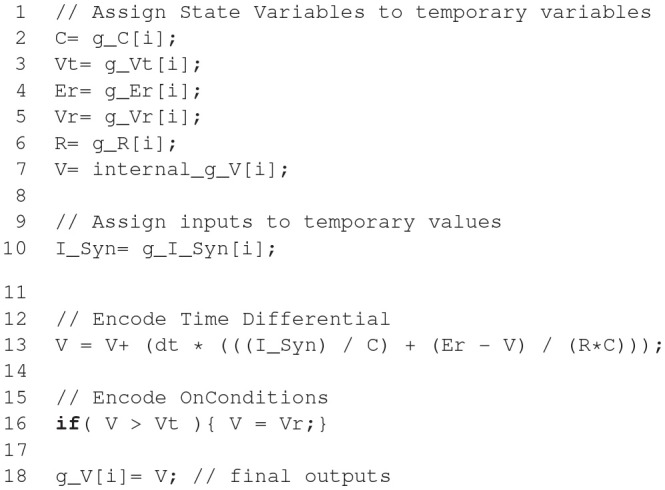
Neurokernel CUDA kernel.

#### 2.8.4. Numerical integration

SpineML does not explicitly solve any equations itself, but allows differential equations to be defined within behavioral regimes. The Experimental layer allows the definition of a preferred integration method to be used to solve these, but does not impose any specific implementation. If simulators do not support the defined integration scheme, it is anticipated that runtime warning should be raised, and a default integration scheme should be used as a fall back. All current simulators support forward Euler integration.

### 2.9. SpiNNaker

The SpiNNaker toolchain differs from the other tools described in this article in that it does not run on general purpose hardware, but only supports the SpiNNaker neuromorphic hardware system as a simulation backend (Furber et al., 2013). The SpiNNaker software is open source and freely available. Its most recent release is version 4.0.0 (Stokes et al., 2007a) which has documentation on how to add new neuron models and new plasticity rules (Stokes et al., 2007b). The SpiNNaker software will run on any Python 2.7 installation, but requires access to a SpiNNaker hardware platform. Free access to a large-scale SpiNNaker machine is possible via the collaboration portal of the Human Brain Project (see section 3).

#### 2.9.1. Main modeling focus

All versions of the neural software supported on SpiNNaker expect users to describe their spiking neural networks using PyNN (Davison et al., 2009), which is then translated automatically into distributed event-driven C code running on the SpiNNaker hardware platform. The degree of code generation within SpiNNaker software is limited to the compilation of the PyNN network description to generate the neural and synaptic data structures for each core to execute. The models themselves are written in hand-crafted C code for the SpiNNaker platform, and attempt to balance a reasonable trade-off between: numerical accuracy, space-utilization and execution efficiency. To support this, Python classes translate the appropriate parameters between the user script and the platform data structures, including the reading back of results.

The decision to support hand crafted code results partly from the structure of the PyNN language which enforces a basic set of neuron and synapse models that end users can use to describe their spiking neural networks, and therefore hand crafting the code that represents these neuron models and synapses makes a sensible starting point. The other reason for supporting hand crafted code is the time required to build a software system for translating general differential equations into code that is small, fast and accurate enough to run on the platform, particularly noting the lack of a floating point unit on the processor. The current toolchain has been in existence for nearly five years and handles the entire process of mapping, running and extracting data from a spiking neural network that could potentially consist of up to one billion neurons and one trillion synapses on a unique architecture and therefore hand crafted code was the simplest approach to execute.

Currently if an end-user requires a neuron model outside those supported by PyNN or one that is not currently implemented in the SpiNNaker software support for PyNN, it will need to be hand crafted. This consists of writing both a Python class, a C code block that can update the state of the new neuron model or synapse on a time-step basis, and finally a *Makefile* that joins the components together to represent the new neuron model. The SpiNNaker software stack currently supports the following PyNN models: *IfCurExp, IfCondExp, IfCurDuelExp, IzhikevichCurExp, IzhikevichCondExp, SpikeSourceArray*, and *SpikeSourcePoisson*.

**Figure 12:**
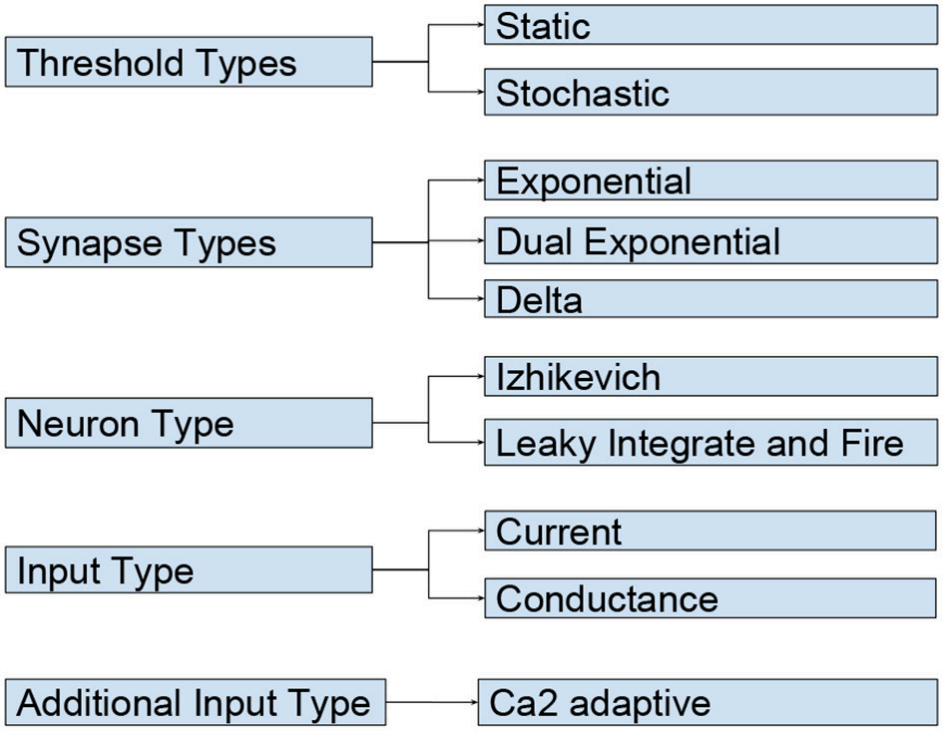
SpiNNaker Python model components. The *threshold types* govern logic for determining if the neuron should spike given a membrane potential; the *synapse type* describes how the weight from a synapse changes over time; the *input type* governs the logic to change from a weight to current; an *additional input type* allows the addition of more current to a neuron given a membrane potential; the *neuron type* encapsulates the equations for processing the current and determining the membrane potential at each time step.

The Python class is used to explain to the SpiNNaker software what SpiNNaker hardware resources the model will require and any parameters needed by the C code representing the model to run on the SpiNNaker platform. The way the Python class describes its requirements is through a set of components, each of which have parameters that need to be transferred to the executable C code and therefore require some memory to store. Each component represents a different part of the overall logic required for a neuron model. The components currently available from within the SpiNNaker software stack are shown in Figure 12. According to that figure, a *IfCurExp* model contains a static *threshold type*, an exponential *synapse type*, a leaky integrate-and-fire *neuron type*, a current based *input type* and no *additional input type*.

Each Python component requires some functions to be implemented for the tool chain to be able to use it. For a *threshold type*, for example, it needs to fill in a function called get_threshold_parameters() which returns a list of parameters needed by the C code for it to execute the neuron model.

The C code used to represent the PyNN neuron model is also split into the same component types as the Python class, but whereas the Python class is used to define what resources were to be used and what parameters are needed by the C code, the C code interfaces require C code functions to be implemented which are used by the boiler plate code that ties all the components together, whilst also handling the event driven nature of SpiNNaker C code.

From the end user's perspective, adding a new neuron model requires the creation of new components of the same types required in the Python class and filling in the functions required by that component. For example, a new *threshold type* in the C code would require a C code which fills in the following functions and structures:

- The threshold_type_t struct, which contains the parameters in the order the Python component listed them.
- The threshold_type_is_above_threshold() function, which has a neuron membrane potential and the threshold_type_t structure for the given neuron as inputs and should return a Boolean dictating if the neuron has spiked given the inputs.

Finally, the end user needs to fill in a template *Makefile* which compiles the C components into executable C code that can run on the SpiNNaker platform. An example is shown in Listing 5 where the components *NEURON_MODEL_H, INPUT_TYPE_H, THRESHOLD_TYPE_H, SYNAPSE_TYPE_H* represent the same components discussed previously and the *SYNAPSE_DYNAMICS* represents the type of logic used for learning (or if the synapses supported are to be static).

**Listing 5:**
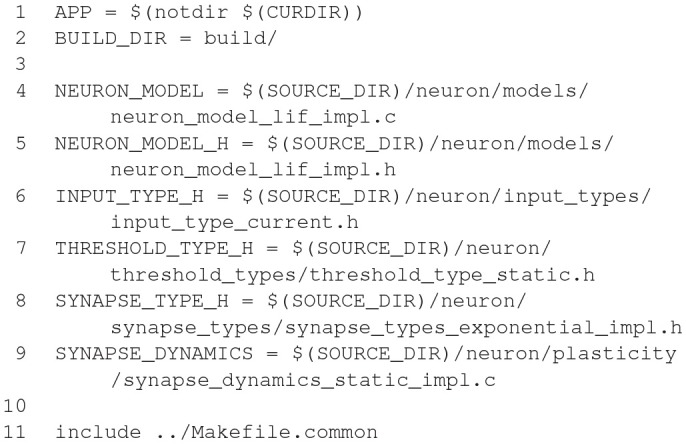
The IfCurExp Makefile for SpiNNaker.

#### 2.9.2. Code generation pipeline

The simulation description consists of a collection of PyNN *Populations* and *Projections*, where *Populations* represent a collection of neurons of a given *model_class*, that embodies a specific neuron model and synapse type that itself embodies a specific set of equations. For example, the PyNN *IfCurExp* model embodies the mathematical equations for a leaky integrate-and-fire neuron (Gerstner and Kistler, 2002) with instantaneous-rise-exponential-decay synapses. The *Projections* represent the physical synapses between neurons of two populations.

New models therefore are represented by a new type of *Population* and the SpiNNaker software supports a template for creating a new neuron model and how to add this into a standard PyNN script (Rowley et al., 2017).

**Figure 13:**
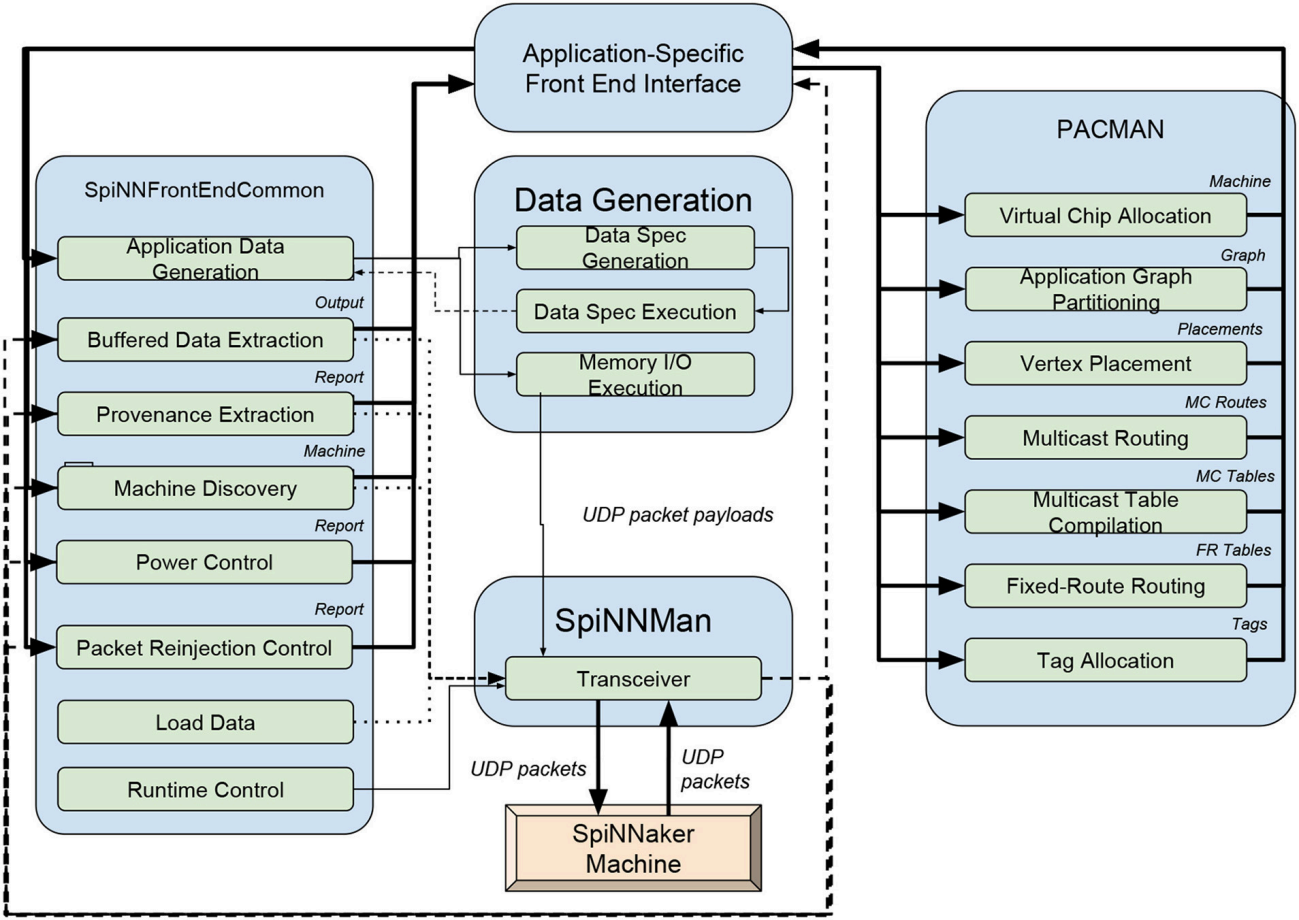
The SpiNNaker software flow. The system starts by utilizing a PyNN script, which is then mapped onto SpiNNaker core sized chunks which are placed and routed on the SpiNNaker machine. The neuron parameters, synapse data, and binaries are loaded onto the machine and executed, with host based runtime functionality to support the executing simulation.

In terms of data and execution, a SpiNNaker simulation consists of a set of distinct stages as shown in Figure 13, and described here (a more detailed description of these stages can be found in Stokes et al., 2007a):

1. The PyNN script description of the neural network is converted into a graph where each vertex contains a number of neurons/atoms, referred to as an *application graph*.
2. The software then maps the application graph onto the SpiNNaker machine, which in itself consists of a set of operations:
  - The application graph is converted into processor sized chunks, referred to as a *machine graph*, where each vertex can be executed on a SpiNNaker processor.
  - The mapping phase decides which SpiNNaker processor will execute each *machine vertex*.
  - The mapping phase continues with allocating routing keys to each neuron that can spike during the simulation. This is used by the router on the SpiNNaker chip to determine where each packet is to be sent.
  - For the packets from neurons a path to take through the SpiNNaker machine is computed, ensuring that each spike packet reaches all of the destination neurons to which it is connected.
  - The routing paths and the routing keys generated are converted into the routing table rules needed by each router on the SpiNNaker machine to ensure the packets are sent to the correct locations.
  - Chips that have a direct connection back to the host machine are configured to control the communication of spikes back to the host, if required.
3. The parameters needed by the neuron models are collected and written down to the memory on the SpiNNaker chips.
4. The compiled executable files that represent the neuron models are loaded along with the router data and the tag information. This stage also handles the control logic that ensures the simulation only runs for the requested duration, and ensures that all the data can be recorded without running out of SDRAM on the SpiNNaker chips by periodically pausing the simulation and extracting the recorded data.
5. The remaining result data and provenance data are extracted from the SpiNNaker machine, and handed back to the PyNN script where the end user can process the results, or change parameters before continuing the execution for a further period. The provenance data is used by the SpiNNaker software to verify that the simulation completed without any issues (such as dropped packets within the communication fabric, or if the simulation lost synchronization), and if any issues were detected, these are reported to the end user.

#### 2.9.3. Numerical integration

The SpiNNaker software framework does not currently provide any support for solving differential equations. Instead, the user must provide C code that updates the state of each neuron at each time step based on the state at the previous time step. The neuron is broken down in to component parts, allowing the combination of various existing components, making the development effort easier. The components are:

1. **The synapse type**. This component controls the flow through the synapses of the neuron. The user can define state variables for each “synapse type” that they wish to define; for example this might include an “excitatory” and an “inhibitory” synapse. This component is called once per time step to: add in the combined weight of several spikes that have been received at each synapse type; to update any state; and finally to read the combined excitatory and inhibitory synaptic contributions to the neuron at the current time step.
2. **The input type**. The main purpose of this component is to convert the synaptic input received from the synapse type component into a current, optionally using the membrane voltage of the neuron. This is usually chosen to be either “current” (in which case the value is just passed on directly) or “conductance” (which makes use of the membrane voltage), but it can be changed to other things depending on the need of the user.
3. **The neuron model**. This component controls the internal state of the neuron body. At each time step, this receives the excitatory and inhibitory currents, as converted by the input type component, and updates its state. The neuron model supports being asked for its membrane voltage (which is used for recording the state, as well as for passing on to the other components). Note also that the neuron model is told when it has spiked, and does not determine this internally (see below). At this point it can perform any non-linear updates as determined by the user.
4. **The threshold model**. This component uses the membrane voltage as generated by the neuron model to decide whether the neuron has spiked. This could for example be simply a static value, or it could be stochastic.

For a discussion on the solving of differential equations within the fixed-point numerical framework available on SpiNNaker (Hopkins and Furber, 2015). Once the user has written their components, they then write a Makefile which combines these with the rest of the provided neuron executable, as shown in Listing 5; this handles the rest of the processing required to execute the neuron model, such as the sending and receiving of spikes, the recording of variables and spikes, as well as handling any plasticity. Spike Time Dependent Plasticity rules can also be generated by the user by providing timing update rules (such as a Spike Pair rule which uses the time between pairs of pre- and post-synaptic spikes to determine how much the synaptic weight is to change) and weight update rules (such as additive, where a fixed value is added to or subtracted from the weight, or multiplicative where the existing weight is taken into account). This splitting again allows an easy way to combine the various components through the use of a common interface.

Though the components of the SpiNNaker neuron software make it easy to combine components, they do also somewhat restrict the rules that can be written to the component interfaces. Thus we are planning on providing a more general framework for the neuron models and plasticity models that allows the combination of the components internally; we will then also provide an packaging which still supports the existing component model to ensure that existing components still work. The more general framework will make it easier to support code generation, as the rules will not generally be split into the components in this fashion.

The general interface for describing neuron models will utilize differential equations, such as that provided by Brian (see section 2.1; Goodman and Brette, 2008, 2009). Initially this would provide support for general linear systems, and the Adaptive Exponential model only. The reason for adopting this position is that SpiNNaker-1 has the limitation of expensive division and integer (or fixed-point) arithmetic only; both of these problems are eliminated in the new SpiNNaker-2 hardware, which is based on the ARM Cortex-M4F core, and thus has hardware support for single precision float and both floating-point and integer division.

The obvious approach to linear ODE systems is to reduce the equations to *Matrix Form*. For example, having the system of equations:

$$\begin{array}{lll} \frac{dv}{dt} & = & a_{\left(0,0\right)}v+a_{\left(0,1\right)}u+b_{0}\\ \frac{du}{dt} & = & a_{\left(1,0\right)}v+a_{\left(1,1\right)}v+b_{1} \end{array}$$

allows to express this in matrix form as:

$$\dot{x}(t)=\begin{array}{l} Ax(t)+b \end{array}$$

where

$$\begin{array}{l} A=\left(\begin{array}{cc} a_{\left(0,0\right)}v & a_{\left(0,1\right)}\\ a_{\left(1,0\right)} & a_{\left(1,1\right)} \end{array}\right)\;b=\left(\begin{array}{c} a_{0}\\ a_{1} \end{array}\right)\;x\left(t\right)=\left(\begin{array}{c} v\left(t\right)\\ u\left(t\right) \end{array}\right) \end{array}$$

With this formulation the forward evolution of the system at time *t* can be expressed as:

$$\begin{array}{l} x\left(t\right)=e^{tA}x_{0}+t\phi_{1}\left(tA\right)\left(b\right) \end{array}$$

where $\phi_{1}\left(A\right)=\left(e^{A}-I\right)A^{-1}$ and *x*_0_ = *x*(0). These matrix exponential calculations can be performed on the host computer using the SciPy routine scipy.linalg.expm, provided that the coefficients in the ODE system remain fixed and that they are not subject to user modification part way through a simulation.

Actual SpiNNaker execution of the solver is a simple matrix multiplication as shown above. It can be performed as a series of fused-multiply-adds. On SpiNNaker (both SpiNNaker-1 and SpiNNaker-2) this can be done with with 32 × 32 operations using internal 64 bit accumulators. The key challenge on the current SpiNNaker hardware is to solve non-linear systems using a minimal use of division and only a limited dynamic range for the variables of the ODE system so that the algorithms do not step outside of the range of the fixed-point number system.

### 2.10. TVB-HPC

The Virtual Brain (TVB, Sanz Leon et al., 2013) is a large-scale brain simulator programmed in Python. With a community of thousands of users around the world, TVB is becoming a validated, popular and standard choice for the simulation of whole brain activity. TVB users can create simulations using neural mass models which can produce outputs for different analysis modalities. TVB allows scientists to explore and analyze simulated and experimental data and contains analytic tools for evaluating relevant scientific parameters in light of that data.

#### 2.10.1. Main modeling focus

Neural mass models (NMMs) are mathematical tools for describing the ensemble behavior of groups of neurons through time. These models contain a set of internal states which describe the system and a set of coupled differential equations which define how the states of the system evolve. An instance of these models in TVB and their implementation is called a “node.” The model output consists of a set of observables identifying states of interest for other nodes. Nodes are linked to each other using a coupling function. This coupling defines the effect of input coming from other nodes. Usually the coupling involves weighting the incoming signals by a factor and then applying a simple function. The weights for coupling may be derived from probabilistic tractography and the diffusion-weighted MRI images of an individual.

Certain system observables can be post-processed to produce simulated BOLD, EEG or EMG signals, among others. These signals can be fed into an analysis step where a measure of system “fitness” with respect to an empirical signal is computed. The number of open degrees of freedom of the NMMs generates a vast parameter space to explore if one wants to fit the model parameters to a specific output. The nature of this workflow enables the iterative modification and exploration of parameters in this admissible space. The problem is embarrassingly parallel (computationally) with respect to the parameter sets to be explored and can be highly parallelized with respect to the node computation for most NMM kernels. Adaptive approaches can be used to optimize the behavior of the models with respect to fitness functions which can relate to the essential characteristics of the higher level signals. Fitness functions can incorporate extended aspects of empirical data, enabling inference of neural mass model parameters through exploration of parameter space.

**Figure 14:**
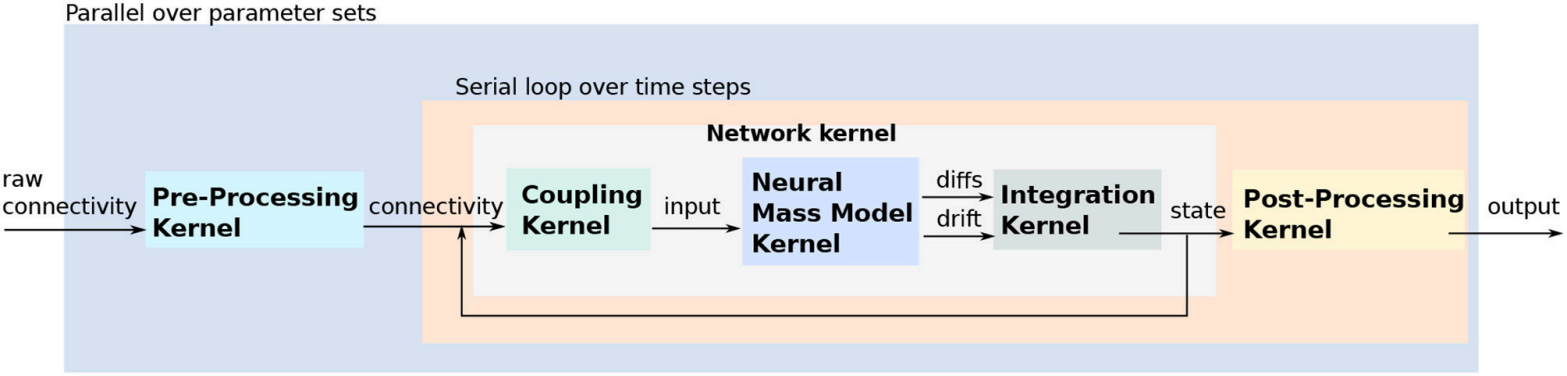
Interaction between the different computational stages in a neural mass model simulation. The raw connectivity from sources such as diffusion tensor imaging is pre-processed to produce a connectivity map between brain regions defined by a parcellation scheme. The connectivity map is fed into a network kernel composed of a coupling, neural mass and integration kernel. The coupling kernel combines the simulated system's current state with the connectivity data to compute the input to each node for the current time step. For each node, the NMM kernel computes the state changes which are fed into the integration kernel to compute the final state at the end of the current time step. The resulting observables are fed back to the coupling kernel and forward to a post-processing kernel to compute derived signals such as BOLD or EEG data for comparison to experimental results. Since there are no dependencies between distinct instances of the network kernel, this data flow can be parallelized over each set of model parameters.

A general description of the simulation can be seen in Figure 14.

The current implementation of TVB is written in Python using NumPy with limited large-scale parallelization over different paramaters. The objective of the TVB-HPC project is enable such large-scale parallelizating by producing a high-level description of models in all stages in the simulation workflow which can then be used to automatically generate high-performance parallel code which could be deployed on multiple platforms. In particular, this allows reifying data flow information. With this approach, neuroscientists can define their pre-processing kernels, coupling, neural mass models, integration schemes, and post processing kernels using a unique interface and combine them to create their own workflows. The result is a framework that hides the complexity of writing robust parallel code which can run either on GPUs or on CPUs with different architectures and optimizations from the end user.

#### 2.10.2. Model notation

The TVB-HPC library is written in Python and makes use of a generic set of classes to define models in an agnostic way, independent of the final target implementation.

In additional to predefined models, TVB-HPC has a built in *BaseModel* class for defining neural mass models and a *BaseCoupling* class for defining coupling kernels through inheritance. The *BaseModel* class defines the following set of attributes:

- **State**: Internal states of the model.
- **Auxex**: Auxiliary mathematical expressions which are used for internal calculations in the model.
- **Input**: Input coming from other neural masses into this neural mass.
- **Drift**: A set of equations which evolve the model from a state at time *t*−1 to time *t*.
- **Diffs**: Differentiable variables in the system.
- **Observ**: Transformations of state variables which are defined as observable or coupled.
- **Const**: Constant values specifically defined for a each model.
- **Param**: Parameters provided to an specific model.
- **Limit**: Minimum and maximum within which the state values must be wrapped to ensure mathematical consistency.

A general NMM inherits from the *BaseModel* class.

As an example, the following listing shows the implementation of the widely used Kuramoto (Kuramoto, 1975) and the Hindmarsh-Rose-Jirsa Epileptor (Naze et al., 2015) models from TVB using the TVB-HPC interface. These two models have been chosen due to their differing levels of complexity.

**Figure:**
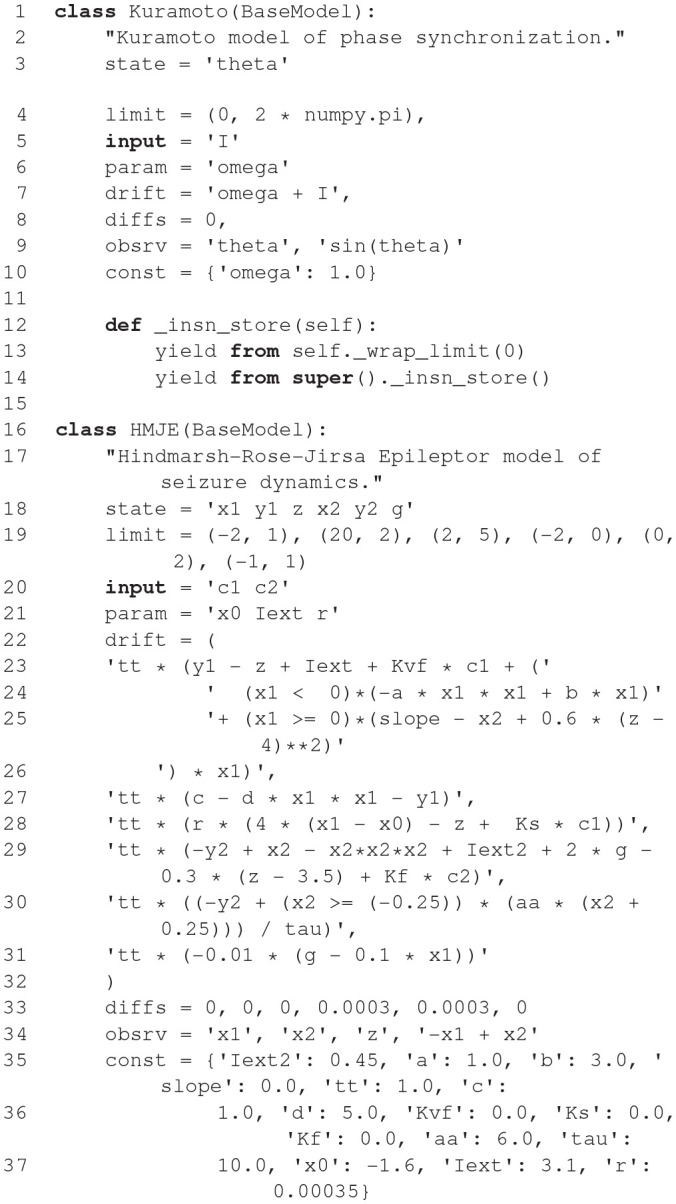


The classes for the coupling kernels are generated in an analogous manner.

#### 2.10.3. Code generation pipeline

Loopy (Klöckner, 2014) is a Python library which aids in the automatic generation of parallel kernels for different target hardware platforms. It includes targets for CUDA, OpenCL, Numba, Numba + CUDA, C, and ISPC. Parallel code in Loopy is generated by enclosing a set of instructions in independent execution domains. The dimensions of a domain is specified using variables named *inames* in Loopy terminology which represent the number of parallel instances that one can process at the same time for the given set of instructions. Notably, Loopy performs and retains explicit data flow metadata about instructions, which enables, for example, nearly automatic kernel fusion. Small, simple kernels can be written and combined using a data flow which defines how variable values are fed from one kernel to the next as input. This allows the creation of complex kernels with assured data dependencies while allowing for unit testing of small component kernels.

Loopy automatically analyzes the data structures and their access patterns within a domain. The user can specify types and ranges for values and access limits to the data structures to control data handling. Loopy assembles the computation within a loop-like environment where each iteration is independent. Code can then be produced for a target platform in the target's specific language.

The *BaseModel* class has functions which translate the information provided in the attributes of a model instance in several steps which ensures the repeatable, coherent and robust generation of code. The steps to follow in order to generate the code are as follows:

1. The kernel's data structures are generated.
2. The kernel domain is defined by setting the limits of the desired *iname* variable. The domain is the main loop within which the parallelization will take place, and the iname is the variable which identifies different instances of parallel executions of this loop.
3. Types for the attributes of the model are specified. Loopy can, in most cases, guess the nature of variables and attiributes, but the user can explicitly state these types as to avoid confusion in the code generation.
4. Expression for constants and distribution of the values for the input, states and parameters are generated.
5. A set of auxiliary expressions which aid the data manipulation inside the kernel may be generated.
6. Expressions to expose and store values for observables (variables which can be accessed after the simulation is done) are generated.
7. Pymbolic (a lightweight SymPy alternative, designed for code generation tasks) is used to translate the set of symbolic expressions representing the drift, diffs, and observables in the next step.
8. The output is wrapped within certain limits to avoid numerical inaccuracies.
9. The final code for a given kernel is generated.

Loopy provides several levels of information about the generated kernel including dependency analysis and scheduling achieved based on the access patterns of the inames to the data structures. An example of the output produced for a test kernel can be seen in the listings below.

**Figure:**
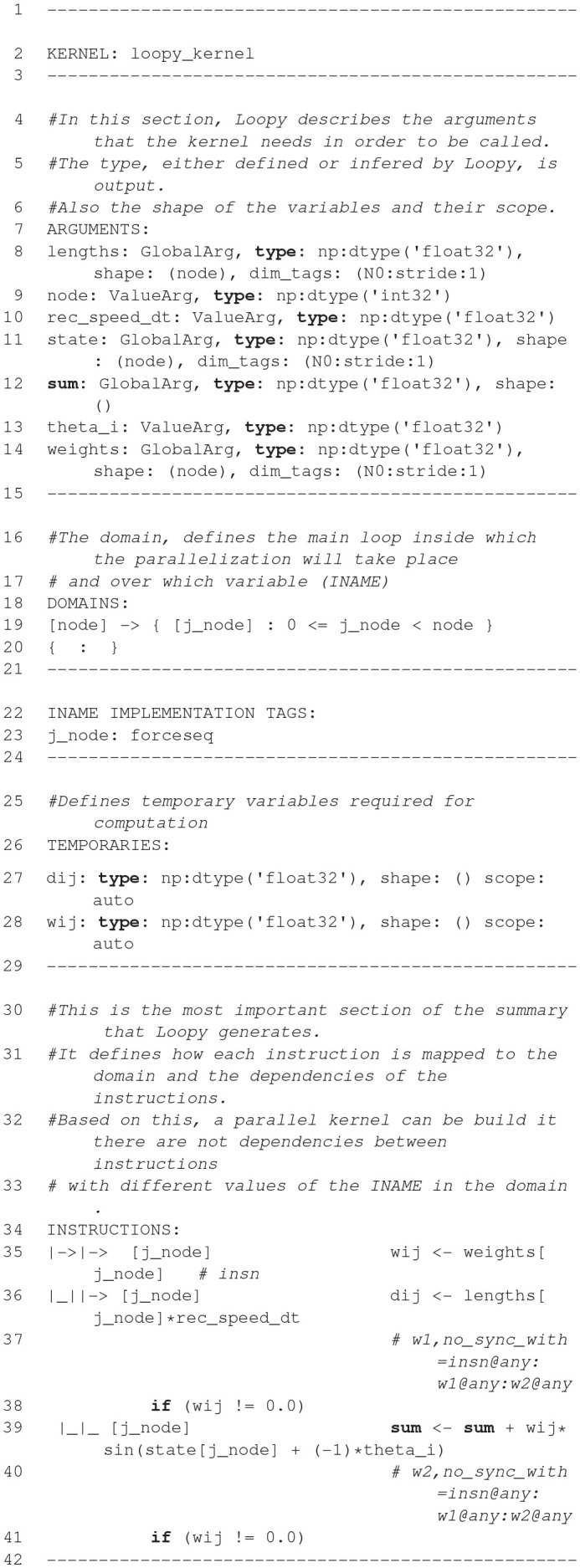


Loopy's debug output elucidate the quantitative kernel analysis. Notably, this includes complete information on the kernel's input (“ARGUMENTS”: datatype, shape, strides), sequence & dataflow of instructions (“INSTRUCTIONS”), as well as temporary variables (“TEMPORARIES”: type, shape, scope of allocation), and finally the loop domains, including their mapping to hardware domains (“INAME IMPLEMENTATION TAGS”) such as local or global work group.

As a concrete use case of TVB-HPC, a kernel which includes the whole workflow described in Figure 14 is presented. The following example shows the generation of a merged kernel including the coupling, the neural mass model and the integration step:

**Figure:**
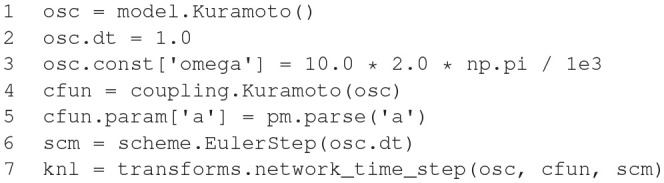


The target code generated for Numba + CUDA:

**Figure:**
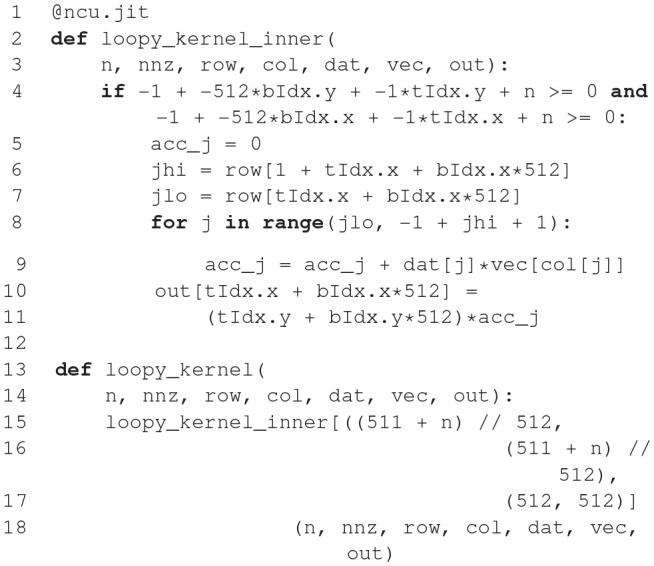


and for Numba:

**Figure:**
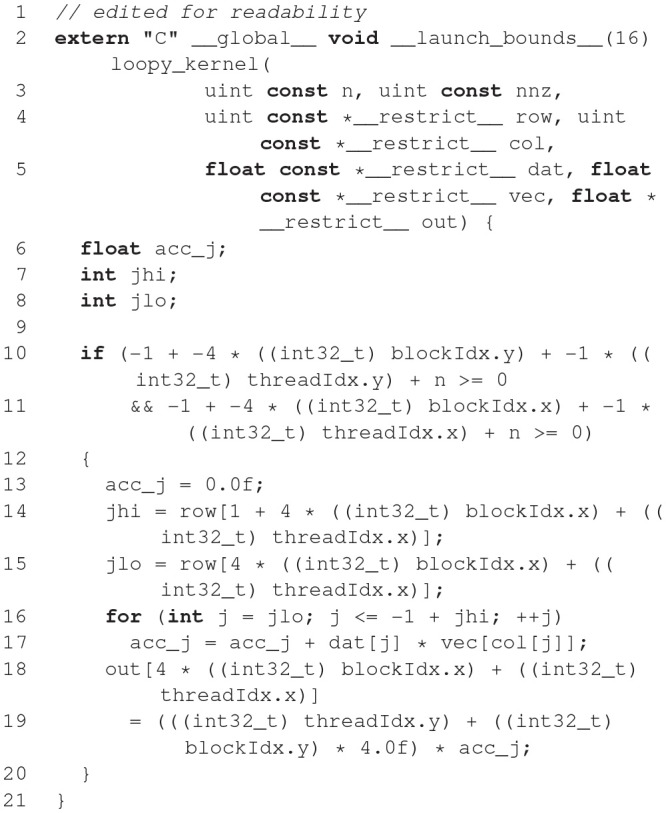


and for CUDA:

**Figure:**
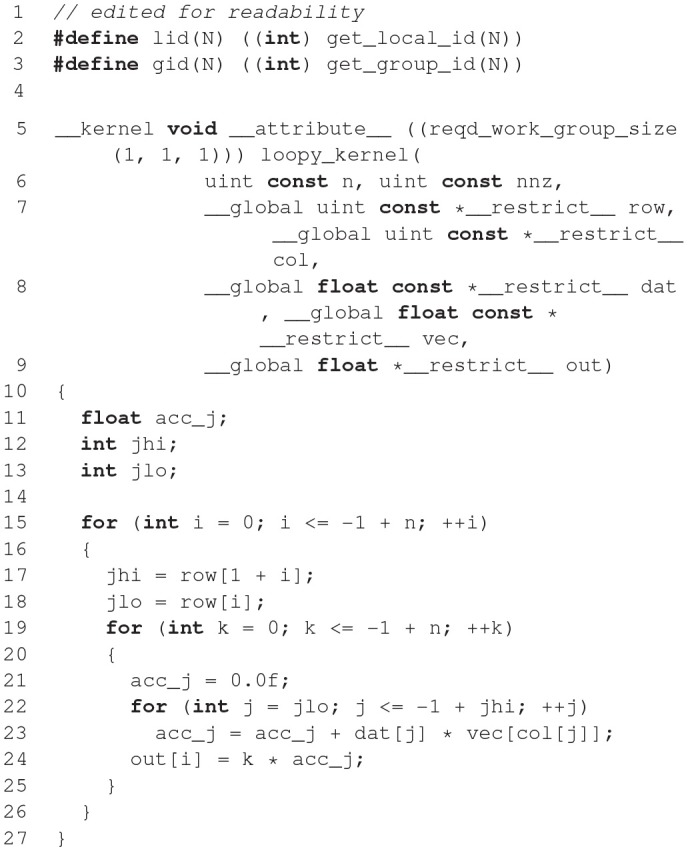


and for OpenCL:

**Figure:**
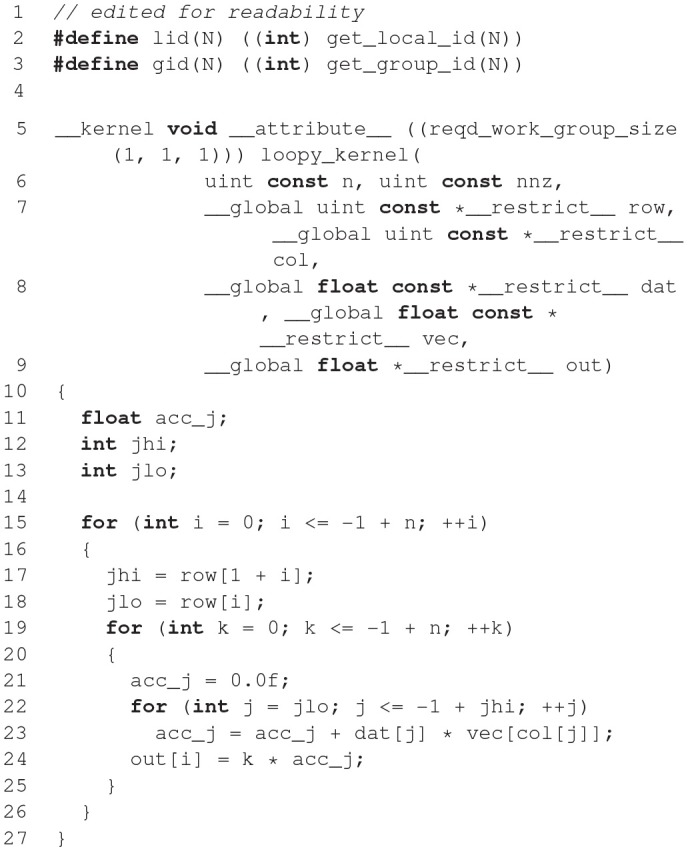


#### 2.10.4. Numerical integration

The ordinary differential equations defined using the BaseModel class (coupling kernels containing only functions) are generally solved using a standard Euler method in TVB-HPC as a proof of concept. It is also possible for a user to define stochastic ODEs. The integration method for those ODEs can be set to the Euler Maruyama method, stochastic Heun or other schemes available in TVB in addition to custom methods provided by the user.

## 3. Hardware and software platforms

All code generation pipelines introduced in section 2 target one or more hardware platforms, on which the generated code can be executed. In this section, we summarize the most prominent hardware platforms and give an overview of collaboration portals, from which the code generation pipelines and the hardware platforms are available with minimal setup overhead for the scientists aiming to use them.

### 3.1. Classical processors and accelerators

Classical von Neumann-architecture computers, dominate the hardware platforms used in the neurosciences. Small spiking neuronal networks or multi-compartmental cells up to a few thousand neurons or compartments are easily simulated on a single central processing unit (CPU) of a modern computer. CPUs allow for maximum flexibility in neural modeling and simulation, but the von Neumann architecture, where instruction fetch and data operation are separated from each other, limits the performance of such a system—this is referred to as the *von Neumann bottleneck*. Even with advanced highly parallel petascale supercomputers available today, the simulation of neural networks are orders of magnitude slower than realtime, hindering the study of slow biological processes such as learning and development.

Graphical processing units (GPUs) are an alternative that can provide better simulation efficiency. A GPU is a co-processor to a CPU, designed to efficiently perform operations that are typical for graphics processing, such as local transformations on large matrices or textures. Because of the structure of graphics operations, it lends itself to a single instruction- multiple data (SIMD) parallel processing approach. Massively parallel GPUs can be repurposed to also accelerate the execution of non-graphics parallel computing tasks, which is referred to as general purpose GPU (GPGPU) computing. The simulation of spiking neuronal networks is well suited for the computing paradigm of GPGPUs, because independent neurons and synapses need to be updated with the same instructions following the SIMD paradigm. However, efficiently propagating spikes in such a network is non-trivial and becomes the main bottleneck for computation performance in large networks (Brette and Goodman, 2012). Implementing the parallelism requires expert knowledge in GPU programming, constituting a large entry barrier for neuroscientists and modelers. After an initial enthusiasm amongst the developers of leading simulators, such as Brian (Goodman and Brette, 2009), GENESIS (Bhalla and Bower, 1993), NEST (Gewaltig and Diesmann, 2007), or NEURON (Hines and Carnevale, 1997), the development of GPU accelerator support has often stalled due to the underlying complexities. Instead, novel GPU based simulators, such as ANNarchy (Vitay et al., 2015), CARLsim (Nageswaran et al., 2009), Cortical Network Simulator (CNS; Mutch et al., 2010), GeNN (see section 2.2 Yavuz et al., 2016), Myriad (see section 2.3 Rittner and Cleland, 2014), NeMo (Fidjeland et al., 2009), were created, each with their own particular focus.

To further accelerate computation and increase efficiency, dedicated hardware architectures beyond the von Neumann model are of interest. Efficiency and flexibility are contrary and cannot both be achieved same time. FPGAs offer a good balance between the generality of CPUs/GPGPUs and physically optimized hardware. An FPGA is a freely programmable and re-configurable integrated circuit device. This paves the way to new computing architecture concepts like dataflow engines (DFE). Following this approach, in principle, application logic is transformed into a hardware representation. In particular, for a neural network simulation, this could be a computation primitive or special function, a neuron model or even an entire neural network or simulation tool (Cheung et al., 2016; Wang et al., 2018). Currently no tools or workflows exist to directly derive an FPGA design from a neural model description. Closing this gap is a topic of research.

Given the multitude of programming paradigms and architectural designs used on modern CPU-, GPGPU-, and FPGA-based systems and their complexity, it is impossible for a novice programmer in the field of neuroscience to write efficient code manually. Code generation is thus often the only way to achive satisfactory performance and system resources utilization.

### 3.2. Neuromorphic hardware

Another approach to surpassing the von Neumann architectures in terms of energy consumption or simulation speed is using physically optimized hardware. For hardware architectures focusing on brain-inspired analog computation primitives the term “neuromorphic” has been coined by Mead (1990). However, nowadays the term neuromorphic computing is used in a much broader sense and also refers to digital systems and even von Neumann architectures optimized for spiking neural networks.

Inspired by the original principle, a large part of the neuromorphic hardware community focuses on physical models, i.e., the analog or mixed-signal implementations of neurons and synapses on a CMOS substrate (cf.Indiveri et al., 2011). Biological observables like the membrane voltage of the neurons are represented as voltages in the silicon neuron implementation and evolve in a time-continuous and inherently parallel manner. One particular example is the BrainScaleS system, which represents a combination of von Neumann and mixed-signal neuromorphic computing principles. Compared to the biological model archetype, the BrainScaleS implementation operates in accelerated time: characteristic model time constants are reduced by a factor of 10^3^−10^4^ (Aamir et al., 2017; Schmitt et al., 2017). In addition, an embedded processor provides more flexibility, especially with respect to programmable plasticity (Friedmann et al., 2017).

Digital implementations range from full-custom circuits, e.g., Intel Loihi (Davies et al., 2018), IBM TrueNorth (Merolla et al., 2014), to optimized von Neumann architectures. One particular example is the SpiNNaker system which is based on standard ARM processors and a highly-optimized spike communication network (Furber et al., 2013). The biggest system constructed to date consists of 600 SpiNNaker boards wired together in a torus shaped network. Each SpiNNaker board contains 48 SpiNNaker chips, where each SpiNNaker chip contains 128 MiB of on-board memory, a router for communicating between chips and up to 18 ARM968E-S (ARM Limited, 2006) processors, each consuming around 1W when all processors are active.

### 3.3. Collaboration platforms

The great variety of code generation pipelines introduced in the previous sections allows neuroscientists to chose the right tool for the task in many modeling situations. However, setting up the pipelines and getting them to play nicely with the different hardware platforms can be a challenging task. In order to ease this task, several collaboration platforms were created in the past years.

The Open Source Brain platform (OSB, http://www.opensourcebrain.org) is intended to facilitate the sharing and collaborative development of models in computational neuroscience. It uses standardized representations of models saved in NeuroML (section 2.5) and shared on public GitHub (https://github.com) repositories to allow them to be visualized in 3D inside a browser, where the properties of the cells and networks can be analyzed.

In addition to viewing the NeuroML models, users who have registered and logged in to the OSB website can run simulations of the models (potentially having edited some of the parameters of the model through the web interface). The NeuroML representation is sent to the OSB server, which uses the code generation options included with the jNeuroML package (section 2.5) to create simulator specific code which can be executed. Currently there are options to execute the model using jNeuroML (limited to point neuron models), the NEURON simulator directly, or in NEURON via the NetPyNE package (http://www.netpyne.org), which allows network simulations to be distributed over multiple processing cores. More simulation platforms are in the process of being added.

The default execution location is to run the simulation on the OSB servers directly, but there is an option to package the simulations and send to the Neuroscience Gateway (NSG, https://www.nsgportal.org) platform. NSG links to the supercomputing facilities of the Extreme Science and Engineering Discovery Environment (XSEDE), and using this option, NetPyNE based simulations can be run on up to 256 cores. The simulation results are retrieved by OSB and can be displayed in the browser without the user needing to access or log in to NSG or XSEDE directly.

The Human Brain Project (HBP) Collaboratory collects the tools developed in the project in one place and allows neuroscientists to organize their work into collaborative scientific workspaces called *collabs*. It provides common web services and a central web-based portal to access the simulation, analysis and visualization software. A central web-accessible storage location and provenance tracking for imported and generated data allow to build on the work of others while making sure that prior work is properly referenced and cited. Moreover, the Collaboratory provides access to the BrainScaleS and SpiNNaker neuromorphic hardware systems and to several European supercomputers, for which users, however, have to provide their own compute time grants.

The main interface to the underlying tools are Jupyter notebooks, which can be used as collaborative workbenches for Python-based scientific collaboration between different users and teams of the system. In addition to interactive instruction, automation and exploration, the notebooks are also used for documentation and to allow neuroscientists to easily share ideas, code and workflows. For live interaction, the system integrates a web chat system.

In the context of the HBP Collaboratory, neuronal description languages and code generation also serve as a means to isolate users from the system in the sense that they can still provide their own model specifications but do not require direct access to compilers on the system. The generation of suitable source code for the target system (i.e., supercomputers or neuromorphic hardware systems) can be handled transparently behind the scenes and the final model implementation can again be made available to all users of the system. Getting access to the HBP Collaboratory requires an HBP Identity Account, which can be requested at https://collab.humanbrainproject.eu.

## 4. Discussion

### 4.1. Summary

The focus of each of the different code generation approaches presented in this article is defined by at least one scientific use case and the supported target platforms. Due to the diversity of requirements in computational neuroscience, it is obvious that there can't be just a single solution which the community would settle on. This review shows that many use cases have already been covered to variable extents by existing tools, each working well in the niche it was created for. As all of the reviewed software packages and tools are available under open source licenses and have active developer communities, it is often easier to extend the existing solutions by support for new use cases instead of creating new solutions from scratch. The following table summarizes the main properties of the different tools:

**Table:** 

	**Models**	**Platforms**	**Techniques**
Brian (2.1)	Point and multicompartmental neurons; plastic and static synapse models	CPUs; GPUs via GeNN	AST transformations; Symbolic model analysis; Code optimization
GeNN (2.2)	Models that can be defined by timestep update code snippet; mostly point neurons and synapses with local update rules	GPUs and CPUs	Direct code generation by a C++ program
Myriad (2.3)	Compartmental neurons; arbitrary synapse models	CPUs; GPUs	Custom object models; AST transformations
NESTML (2.4)	Point neurons	CPUs via NEST	Custom grammar definitions; AST transformations; model equation analysis
NeuroML/LEMS (2.5)	Point and multicompartmental neurons; plastic and static synapse models	CPUs via NEURON and Brian; SBML	Procedural generation; template-based generation; semantic model construction
NineML (2.6)	Models defined by a hybrid dynamical system; mostly point neurons and synapses with local update rules	CPUs via NEURON, NEST and PyNN	symbolic analysis; template-based generation
NEURON/NMODL (2.7)	Point and multicompartmental neurons; plastic and static synapse models; linear circuits; reaction-diffusion; extracellular fields; spike and gap junction coupled networks	CPUs; GPUs via CoreNEURON	Custom grammar; parse tree transformations; GUI Forms
SpineML (2.8)	Models defined by a timestep update code snippet; mostly point neurons and synapses with local update rules; generic inputs support compartments and non-spiking components	CPU via BRAHMS and PyNN; GPU via GeNN and Neuorkernel	XSLT code templates and libSpineML
SpiNNaker (2.9)	Common point neuron models with either static of plastic synapses	SpiNNaker	Hand crafted modular source code, loaded through a complex mapping process from a graph representation
TVB-HPC (2.10)	Neural mass models	CPUs; GPUs	AST transformations

## 5. Conclusion

In order to integrate and test the growing body of data in neuroscience, computational models have become increasingly complex during recent years. To cope with this complexity and unburden users from the task of manually creating and maintaining model implementations, code generation has become a popular approach. However, even though all code generation pipelines presented in this article try to reduce the users' load, they differ considerably when it comes to which aspects they simplify for the user. While, for example, NeuroML (section 2.5) and NineML (section 2.6) aim for simulator independence, and their associated code generation tools do not at present perform heavy transformations on the equations contained in a model specification, NESTML (section 2.4) targets only NEST and analyzes the equations and tries to find the most accurate and efficient solver for them. Myriad (section 2.3) on the other hand has a focus on the automatic parallelization of multicompartment cells on CPU and GPGPU systems but only provides two built-in solvers. The emphasis for Brian (section 2.1) is on the simplest possible user syntax and flexibility of the code generation process (e.g., easily incorporating user-defined functions).

One important use case of code generation in computational neuroscience is the separation of users from the underlying hardware system and corresponding libraries. The fact that platform specific code is generated from a higher-level description instead of directly written by the user allows model implementations to be generated for multiple simulators and certain parts of the execution system to be exchanged without any need for changes in the original model description. On web-based science portals like the Open Source Brain or the Human Brain Project Collaboratory (section 3) this aspect can prevent the execution of arbitrary code by users, which increases the overall security of the systems.

## Author contributions

IB, JE, AM, and DP are the authors of NESTML and have coordinated the work on this article and written the section about NESTML. TN is the creator of GeNN and has written passages on GPU platforms and related simulators. RB, DG, and MS are the authors of Brian and wrote the section on Brian. AS, DL, and AR are the authors of the SpiNNaker software and have written the SpiNNaker section. GT has written passages on classical processors and accelerators, EM has written the passages about neuromorphic hardware. JE and PG wrote the section about collaboration platforms. AD and TGC participated in the development of the NineML specification and the Python NineML library, and wrote the section on NineML, Pype9, and 9ML-toolkit. TGC developed PyPe9. PR, AT, CF, and DC have written the section on SpineML and GPU code generation. PR is an author of SpineML and AT is the author of libSpineML. BM and PG are core NeuroML and LEMS developers, and have written the section on NeuroML/LEMS. MH is the author of NMODL and wrote the NMODL section together with PK who contributes to the development of the code generator for CPU/GPU architectures. MW, SD-P, and AP have written the section on how automatic code generation is being used to enhance the performance of workflows from The Virtual Brain. The final version of the article was written and edited jointly by all authors.

### Conflict of interest statement

The authors declare that the research was conducted in the absence of any commercial or financial relationships that could be construed as a potential conflict of interest.
